# Heterologous DNA Prime- Subunit Protein Boost with Chikungunya Virus E2 Induces Neutralizing Antibodies and Cellular-Mediated Immunity

**DOI:** 10.3390/ijms241310517

**Published:** 2023-06-23

**Authors:** Fernanda Caroline Coirada, Edgar Ruz Fernandes, Lucas Rodrigues de Mello, Viviane Schuch, Gúbio Soares Campos, Carla Torres Braconi, Silvia Beatriz Boscardin, Daniela Santoro Rosa

**Affiliations:** 1 Departamento de Microbiologia, Imunologia e Parasitologia, Universidade Federal de São Paulo (UNIFESP/EPM), São Paulo 04023-062, Brazil; fernanda.coirada@gmail.com (F.C.C.); edgar.ruz.f@gmail.com (E.R.F.); ctbsantos@unifesp.br (C.T.B.); 2 Departamento de Biofísica, Universidade Federal de São Paulo (UNIFESP/EPM), São Paulo 04044-020, Brazil; lucasr.mello@hotmail.com; 3 Departamento de Análises Clínicas e Toxicológicas, Universidade de São Paulo (USP), São Paulo 05508-000, Brazil; vivischuch@gmail.com; 4 Laboratório de Virologia, Universidade Federal da Bahia (UFBA), Salvador 40110-909, Brazil; gubiosoares@gmail.com; 5 Departamento de Parasitologia, Universidade de São Paulo (USP), São Paulo 05508-000, Brazil; sbboscardin@usp.br; 6 Instituto Nacional de Ciência e Tecnologia de Investigação em Imunologia—INCT (III), São Paulo 05403-900, Brazil

**Keywords:** CHIKV, envelope, heterologous prime-boost, adjuvants, DNA vaccine, single chain, recombinant protein

## Abstract

Chikungunya virus (CHIKV) has become a significant public health concern due to the increasing number of outbreaks worldwide and the associated comorbidities. Despite substantial efforts, there is no specific treatment or licensed vaccine against CHIKV to date. The E2 glycoprotein of CHIKV is a promising vaccine candidate as it is a major target of neutralizing antibodies during infection. In this study, we evaluated the immunogenicity of two DNA vaccines (a non-targeted and a dendritic cell-targeted vaccine) encoding a consensus sequence of E2_CHIKV_ and a recombinant protein (E2*_CHIKV_). Mice were immunized with different homologous and heterologous DNAprime-E2* protein boost strategies, and the specific humoral and cellular immune responses were accessed. We found that mice immunized with heterologous non-targeted DNA prime- E2*_CHIKV_ protein boost developed high levels of neutralizing antibodies, as well as specific IFN-γ producing cells and polyfunctional CD4^+^ and CD8^+^ T cells. We also identified 14 potential epitopes along the E2_CHIKV_ protein. Furthermore, immunization with recombinant E2*_CHIKV_ combined with the adjuvant AS03 presented the highest humoral response with neutralizing capacity. Finally, we show that the heterologous prime-boost strategy with the non-targeted pVAX-E2 DNA vaccine as the prime followed by E2* protein + AS03 boost is a promising combination to elicit a broad humoral and cellular immune response. Together, our data highlights the importance of E2_CHIKV_ for the development of a CHIKV vaccine.

## 1. Introduction

Over recent years, chikungunya virus (CHIKV) infection has raised public health concern due to the increasing number of outbreaks around the world and the comorbidities associated with this infection. Chikungunya (CHIK) is an arthropod-borne virus (arbovirus) first isolated in Tanzania [1], transmitted mainly by the *Aedes* species (such as *A. aegypti* and *A. albopictus*) that caused sporadic outbreaks until 1990, along with more relevant episodes over the last 20 years in Africa, India, and Southeast Asia [2], and recently spreading to the Americas [3,4]. Four different lineages were identified: West African, Asian, East-Central-South-African (ECSA), and the Indian Ocean Lineage (IOL) [5]. In Brazil, two different lineages were introduced simultaneously in 2014; the Asian lineage was detected in Oiapoque (Amapá), and 7 days later, the ECSA lineage was detected in Feira de Santana (Bahia) [6]. The molecular genotype surveillance of the circulating strains suggests the predominance of the ECSA lineage and its establishment in Brazil [7]. The ECSA lineage continues to spread across the country through interregional importation events, likely mediated by human mobility [8].

CHIKV is a non-fatal, self-limiting infection characterized by fever, nausea, rashes [9], and polyarthralgia, which can persist for years [10,11,12]. Unfortunately, to date, there are no specifically licensed drugs for treatment or vaccines available to prevent this disease. Currently, control strategies aim to reduce the spread of mosquitoes and human exposure. The development of a vaccine to prevent CHIKV infection is a global health priority, mainly because this disease imposes high costs due to the long-term treatment of arthralgia [13].

CHIKV belongs to the *Alphavirus* genus with a single-stranded, positive-sense RNA genome (approximately 12 kb) encoding nonstructural proteins (nsP1-4) required for virus replication [14] and structural proteins (C-E3-E2-6K-E1) [15]. The structural E1–E2 protein heterodimers, the main component of the virus surface [16], mediate fusion and binding to the target cell membrane [17], respectively. The humoral immune response plays a fundamental role in CHIKV control [18,19,20], and IgG neutralizing antibodies control virus dissemination in animal models [21,22]. The main target of anti-CHIKV antibodies during infection is the E2 glycoprotein [23,24,25], and the passive transfer of E2 polyclonal human immunoglobulin and specific monoclonal antibodies has been successfully used in therapy [26]. Therefore, the E2 glycoprotein is a promising vaccine immunogen.

Over the years, several candidates have been evaluated in clinical trials [27,28] for their use as an effective prophylactic CHIKV vaccine using different platforms, such as inactivated and live attenuated viruses [29], subunit vaccines [30,31], virus-like particles [32] recombinant virus-vectored vaccines [33,34,35], and DNA vaccines [36,37,38]. Unlike inactivated and live-attenuated viruses, subunit vaccines contain specific antigenic fragments, thus eliminating the safety concerns of incomplete inactivation or virulence recovery. Furthermore, subunit vaccines are safe, and can target specific, well-defined neutralizing epitopes with an improved immunogenicity and/or efficacy [39,40]. On the other hand, DNA vaccines are stable, safe, cheap, and easy to manufacture. Although this vaccine platform presents a high safety, the greatest challenge for its clinical use is its poor immunogenicity in humans. Thus, several strategies have been implemented to overcome this caveat and increase immunogenicity, such as design optimization [41], antigen targeting to dendritic cells [42], in vivo electroporation [43,44], and heterologous prime-boost immunization strategies [45].

Antigen targeting to dendritic cells (DC) via the DEC205 endocytic receptor using chimeric monoclonal antibodies has been shown to increase specific immune responses [46,47,48]. This phenomenon was also observed using DNA vaccines encoding single-chain variable fragments targeting to the same DEC205 receptor expressed by CD8α^+^ dendritic cells [49,50,51,52].

In the present study, we investigated the specific humoral and cellular immune responses after the immunization of mice with DNA vaccines encoding E2_CHIKV_ (a non-targeted and a dendritic cell-targeted vaccine) and a recombinant protein (E2*_CHIKV_). Our results showed that mice immunized with the heterologous DNA prime- E2*_CHIKV_ protein boost strategy developed high levels of neutralizing antibodies, as well as specific IFN-γ producing cells and polyfunctional CD4^+^ and CD8^+^ T cells. We also mapped the H-2^b^ most immunogenic epitopes in the E2 protein.

Our results led us to conclude that heterologous DNA vaccine prime- E2*_CHIKV_ protein boost immunization was able to induce efficient humoral and cellular immune responses simultaneously, which is important for infection control.

## 2. Results

### 2.1. Characterization of E2-Based Vaccines

A consensus sequence of the ectodomain of the E2 sequence (aa 1–422) was generated after the alignment of 79 CHIKV isolates with a 94.23% homology between the amino acids. Seventy-five sequences were ECSA and four were Asian genotypes (Table S1), respectively. Of note, none of the 79 sequences analyzed contained the E2V264A mutation that was previously described to enhance fitness in *Aedes aegypti* [53] The transmembrane region was removed (aa 365–422) for the DNA vaccines, while for the recombinant protein, the cysteine N-terminus (aa 1–28) was also removed (Figure S1a). The full length E2 protein contains 17 cysteine residues which increase the intrinsic hydrophobicity of the protein, which results in protein aggregation during the purification steps. Previous studies [54] have shown that the removal of the N-terminus cysteine portion and the transmembrane region could improve the production without compromising the B cell epitope sites.

Subsequently, we cloned the E2 ectodomain sequence (aa 1–364) optimized for mammalian expression in the pVAX1 vector (pVAX-E2) (Figure S1b-I) to generate a non-targeted DNA vaccine, and in the single-chain variable fragment (scFv) αDEC205 vector (scDEC-E2) that targets the E2 antigen to CD8α^+^ DEC205^+^ dendritic cells (DC) to produce a DC-targeted DNA vaccine (Figure S1b-II) [50,55]. Furthermore, in vitro expression of the E2 protein was confirmed after transient transfection of the HEK293T cells. Immunoblotting analysis revealed that both plasmids expressed the recombinant E2 in the supernatant (Figure S1c-I) and cell lysates (Figure S1c-II), respectively.

For recombinant E2* production, we cloned the E2 ectodomain sequence (aa 29–364) optimized for bacteria expression into the pET21a vector (pET21a-E2*). Upon expression, the E2* protein was purified by affinity chromatography. SDS-PAGE analysis under reducing conditions showed that the recombinant E2* displayed the expected molecular weight (~40 kDa) (Figure S1d) and was specifically recognized by the serum from of a convalescent CHIKV^+^ individual (Figure S1e-I), while the serum of a non-infected individual was unable to recognize the protein (Figure S1e-II).

### 2.2. Immunization with E2_CHIKV_-Based Vaccines Induces Robust Specific Humoral Responses

**Figure 1:**
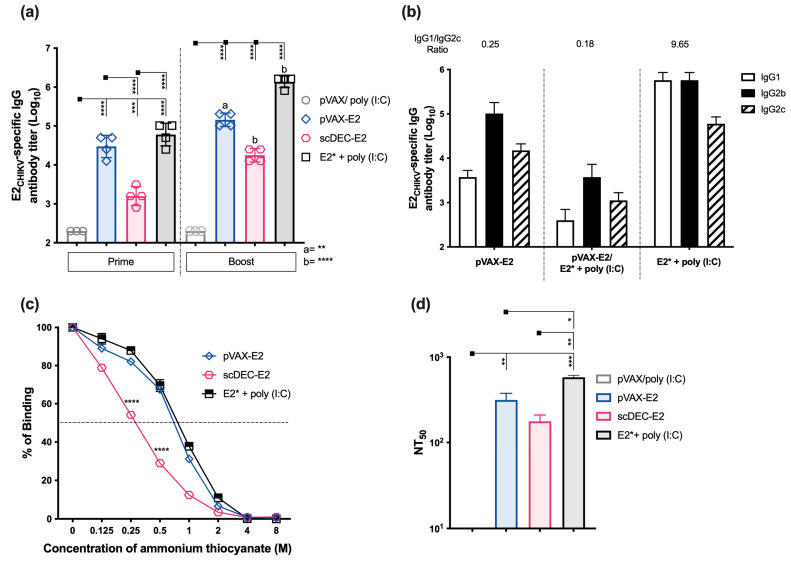
Homologous DNA or E2*_CHIKV_ protein prime-boost immunization induces robust humoral responses. C57BL/6 mice were either immunized intramuscularly twice with 100 μg of pVAX-E2 (non-targeted DNA vaccine), with scDEC-E2 (a DC-targeted DNA vaccine) followed by in vivo electroporation, or with 10 μg of E2* recombinant protein + poly (I:C) subcutaneously (immunization strategy displayed in Figure S2a). The control group received empty pVAX vector and poly (I:C). Blood samples were collected 14 days after each immunization to evaluate the humoral immune response. (**a**) Total E2*-specific IgG titers. (**b**) E2*-Specific IgG subclasses after boost. (**c**) Antibody affinity of pooled mouse sera after incubation with increasing concentrations of ammonium thiocyanate. (**d**) For PRNT, pooled sera were incubated with 100 PFU of CHIKV and the NT_50_ is displayed. Statistical analysis was performed by one-way ANOVA followed by Tukey post-hoc test. Data represent the mean ± SD and are representative from 3 independent experiments. (a,b) statistical significance when compared to the first dose. * *p* < 0.05, ** *p* < 0.01, *** *p* < 0.001, and **** *p* < 0.0001.

To access the immunogenicity of E2_CHIKV_-based vaccines, C57BL/6 mice were immunized twice in a homologous regimen, with a non-targeted pVAX-E2 DNA vaccine, a DC-targeted scDEC-E2 DNA vaccine, or a purified recombinant E2* protein. DNA vaccines were administered intramuscularly in association with in vivo electroporation, whereas the E2* protein subunit vaccine was administered subcutaneously in association with Poly (I:C) as an adjuvant. Control groups received either only pVAX or the adjuvant (immunization strategy displayed in Figure S2a). Fifteen days after the second dose, the pooled sera of all groups (except the control group) recognized the recombinant E2* protein by immunoblotting (Figure S2b). Next, we analyzed the E2-specific IgG titers (Figure 1a). After prime, mice that received the non-targeted pVAX-E2 DNA vaccine or the recombinant protein E2* + poly (I:C) presented similar specific E2-specific titers which were higher than DC-targeted scDEC-E2 DNA vaccine group. After the boost, there was a pronounced increase in the IgG response in all groups, but lower titers were still observed in the DC-targeted scDEC-E2 group. On the other hand, the control group presented negligible antibody titers. Next, we accessed the specific IgG subclasses induced after the boost. Sera of all immunized groups presented IgG1, IgG2b, and IgG2c subclasses (Figure 1b), and we observed the lowest IgG1/IgG2c ratio in groups immunized with both DNA vaccines (pVAX-E2 and scDEC-E2), thereby suggesting a Th1-biased response. We also assessed antibody affinity, and antibodies from all groups presented a similar affinity except for the scDEC-E2 group (Figure 1c). The quality of the generated antibodies was evaluated by the plaque reduction neutralizing assay (PRNT) and sera from all experimental groups were able to neutralize CHIKV infection (Figure 1d). Immunization with recombinant E2* displayed a superior neutralizing ability with the highest NT50 value when compared to all other groups that received the DNA vaccination (pVAX-E2 and scDEC-E2). Sera from the control groups did not present a significant neutralizing ability.

Finally, we evaluated the persistence of the antibody response 45 days after the boost dose. Similar antibody titers were displayed compared to those obtained 15 days after the boost (Figure S3a) and antibody affinity also showed similar levels (Figure S3b). Notably, the neutralization ability remained preserved 45 days after the boost in all groups (Figure S3c).

### 2.3. E2_CHIKV_ T Cell Epitope Mapping

**Figure 2:**
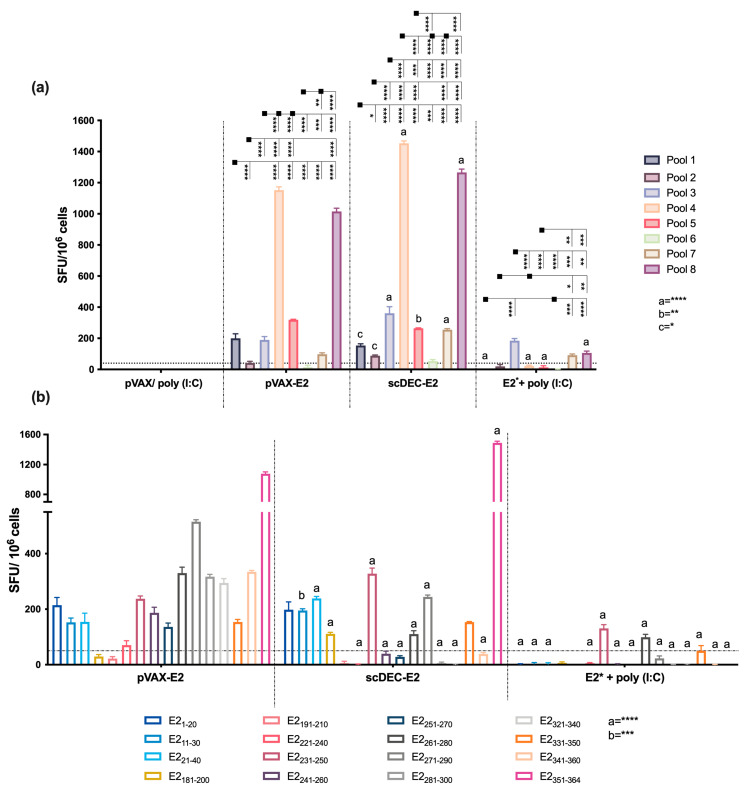
Homologous immunization elicits IFN-γ-producing cells against specific epitopes. C57BL/6 mice were immunized as described in Figure 1 (immunization strategy displayed in Figure S2a). Fifteen and forty-five days after the boost, the number of IFN-γ-producing cells was assessed with ELISpot assay. (**a**) Splenocytes were cultured for 18 h in the presence of E2-pooled peptides (10 μg/mL) organized as a matrix. (**b**) Forty-five days after the immunization, splenocytes were cultured in the presence of 16 individual peptides (10 μg/mL) under similar conditions. SFU: spot forming units. a,b,c: statistical analysis compared to the pVAX-E2 group. Statistical analysis was performed by Two-way ANOVA followed by Bonferroni post-hoc. Data represent mean ± SD and are representative of 2 independent experiments. (a,b,c), statistical significance when compared to the pVAX-E2 group. * *p* < 0.05, ** *p* < 0.01, *** *p* < 0.001, and **** *p* < 0.0001.

We investigated the ability of the vaccine candidates to elicit broad cellular immune responses. To this end, a peptide library containing the E2_CHIKV_ envelope protein was synthesized (Table S2), and the peptides were organized in an optimized matrix (8 pools containing 9 peptides each Table S3). Mice were immunized as previously described (Figure S2a), and fifteen days after boost, splenocytes were obtained and IFN-γ production was assessed by ELISpot (Figure 2a). Splenocytes from mice immunized with both DNA vaccines (pVAX-E2 or scDEC-E2) presented the highest number of IFN-γ-producing cells directed to pools 4 and 8, with a higher magnitude observed after immunization with scDEC-E2. We also detected a response against pools 1, 3, 5, and 7, albeit with a lower magnitude in groups immunized with the DNA vaccines. Regarding IFN-γ-producing cells induced by recombinant E2* protein immunization, we observed the lowest response. In contrast, the control group presented a negligible specific response. Using the Deconvolute This! software (v. 2.0), we selected 16 potential immunogenic peptides for further evaluation with the ELISpot assay (Figure 2b). Immunization with pVAX-E2 induced IFN-γ producing cells against 14 of the16 tested peptides (E21-20, E211-30, E221-40, E2221-240, E2231-250, E2241-260, E2251-270, E2261-280, E2271-290, E2281-300, E2321-340, E2331-350, E2341-360, E2351-364). Furthermore, mice that received scDEC-E2 presented IFN-γ responses against nine peptides. However, the immune response induced by E2* immunization was primarily directed against only three peptides (E2_231–250_, E2_261–280_, and E2_331–350_, respectively).

We also characterized the antigen-specific CD4- and CD8^+^ T cells that produce IFN-γ and TNF-α. We pulsed the splenocytes with six peptides (E2_1–20_, E2_21–40_, E2_231–250_, E2_261–280_, E2_271–290_, and E2_351–364_, respectively), which were selected based on their ability to induce a strong IFN-γ response in the different groups. Our results revealed a higher frequency of CD4^+^ or CD8^+^ T cells (Figure S4) that were able to produce IFN-γ, TNF-α, or both cytokines in splenocytes from the mice that were immunized with both DNA vaccines. On the other hand, immunization with the recombinant E2* + poly (I:C) group induced lower cytokine production. These data suggest that the DNA vaccines demonstrated a superior ability to induce cellular immune responses compared to homologous immunization with the E2* protein. Additionally, the performance of the non-targeted DNA vaccine pVAX-E2 was determined to be superior to the DC-targeted scDEC-E2 DNA vaccine.

### 2.4. The Heterologous DNA Prime-E2*_CHIKV_ Protein Boost Strategy Induces Both Humoral and Cellular Immune Responses

Next, we performed a systematic comparison of the immune responses induced by the homologous and heterologous DNA prime-protein boost strategies. We compared the homologous and heterologous prime-boost strategies using DNA vaccines (pVAX-E2 or scDEC-E2) as the prime followed by recombinant E2* protein + poly (I:C) adjuvant as the boost. Initially, the immunofluorescence assay showed that sera from all immunized mice recognized Vero E6 cells infected with CHIKV (Figure S5a). In contrast, antibodies from the control group (pVAX/poly (I:C)) were unable to recognize CHIKV-infected cells. In addition, phalloidin staining of cell cytoskeletons confirmed that CHIKV was recognized within the cells by sera from the immunized mice (Video S1) but not by the control group (Figure S5b).

**Figure 3:**
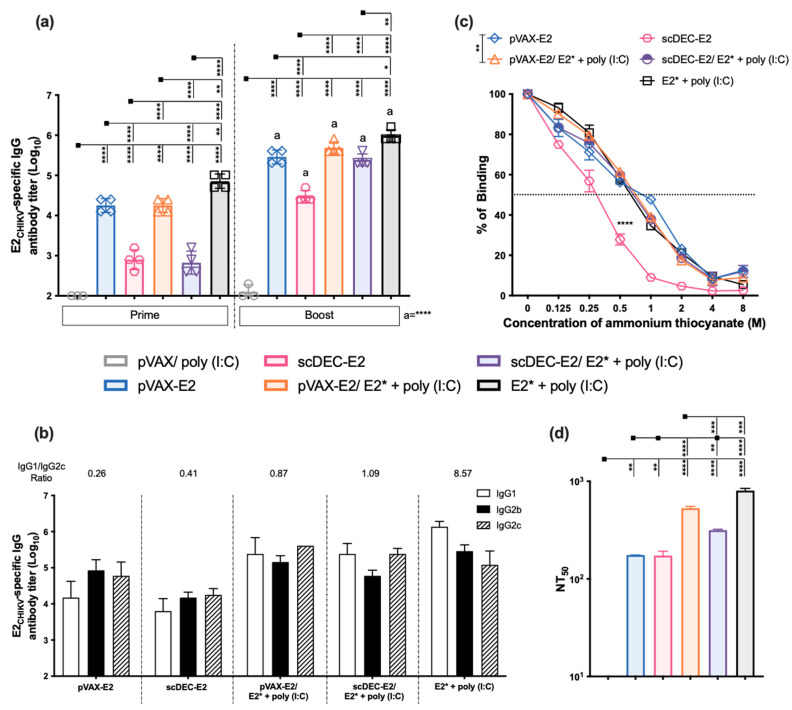
Immunization with vaccines encoding E2_CHIKV_ induces robust humoral immune responses. C57BL/6 mice were either immunized intramuscularly twice with 100 μg of pVAX-E2 (non-targeted DNA vaccine), with 100 μg of a DC-targeted scDEC-E2 DNA vaccine followed by in vivo electroporation, or with 10 μg of E2* recombinant protein + poly (I:C) subcutaneously (immunization strategy displayed in Figure S2a). For the heterologous DNA prime-protein boost, mice received one dose of a DNA vaccine (pVAX-E2 or scDEC-E2) followed by a boost with E2* recombinant protein + poly (I:C). Blood samples were collected 14 days after each immunization to evaluate the humoral immune response. (**a**) Total E2*-specific IgG titers. a- statistical analysis in comparison to the first dose. (**b**) E2*-specific IgG subclasses after the boost. (**c**) Antibody affinity of pooled mouse sera after incubation with increasing concentrations of ammonium thiocyanate. (**d**) For PRNT, pooled sera were incubated with 100 PFU of CHIKV and the NT_50_ is displayed. Statistical analysis was performed with the one-way ANOVA followed by the Tukey post-hoc test. Data represent the mean ± SD and are representative of 2 independent experiments. (**a**) Statistical significance when compared to the first dose. * *p* < 0.05, ** *p* < 0.01, *** *p* < 0.001, and **** *p* < 0.0001.

Subsequently, we analyzed E2-specific antibody titers using ELISA (Figure 3a). After the prime, the groups that received scDEC-E2 were found to develop the lowest responses. After the boost, the highest titers were reached in the group receiving homologous recombinant E2* protein + poly (I:C), while the lowest response was detected in the homologous scDEC-E2 group. In heterologous groups, the comparison between the pVAX-E2 and scDEC-E2 groups showed similar antibody levels. Furthermore, after the boost, we detected all the IgG subclasses that were analyzed (Figure 3b). As previously observed, homologous DNA immunization (scDEC-E2 or pVAX-E2) showed the lowest IgG1/IgG2c ratio. Antibody affinity analysis (Figure 3c) demonstrated similar patterns except for the scDEC-E2 group, which presented a slightly lower affinity as previously observed. Notably, heterologous immunization with scDEC-E2/E2* + poly (I:C) greatly improved affinity. Next, we examined the neutralization profile (Figure 3d) and observed that the homologous E2* protein+ poly (I:C) group presented slightly higher NT_50_ titers than the heterologous DNA prime- E2* protein boost groups. The homologous DNA regimen for both the non-targeted pVAX-E2 and DC-targeted scDEC-E2 groups presented the lowest NT_50_ titers. These results led us to conclude that homologous recombinant E2* protein immunization induced the most robust humoral immune response followed by the heterologous DNA prime- E2*protein boost regimens.

**Figure 4:**
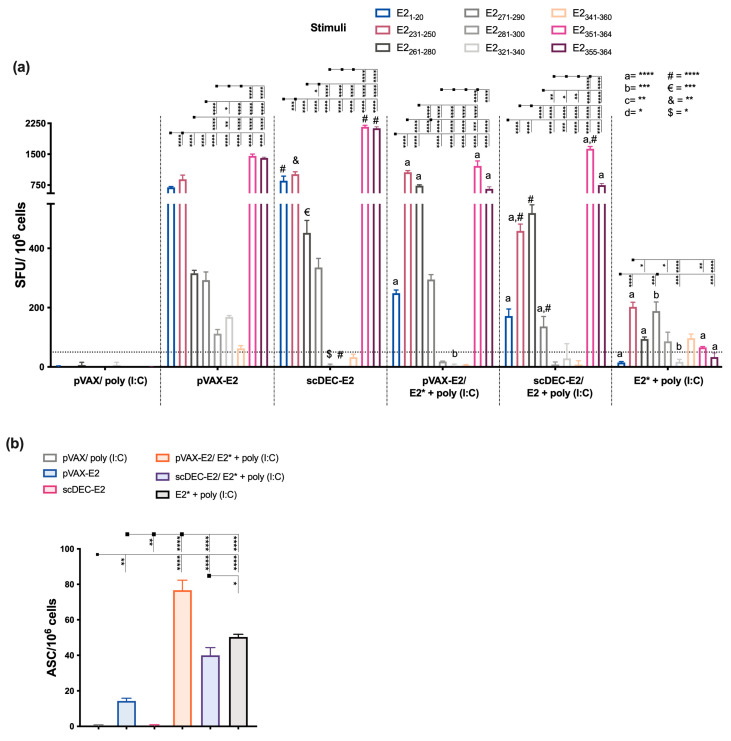
Immunization with vaccines encoding E2_CHIKV_ elicits robust T and B cell responses. C57BL/6 mice were immunized as described in Figure 3 (immunization strategy displayed in Figure S2a). (**a**) Fifteen days after the boost, spleen cells were cultured in the presence of individual peptides from the E2* recombinant protein (10 μg/mL) to evaluate the number of IFN-γ-producing cells using the ELISpot assay. SFU: spot forming units. a, b, c, d represent statistical significance between the homologous and respective heterologous prime-boost strategies. #, €, &, $ represent the statistical significance between the pVAX-E2 and scDEC-E2 groups in the homologous immunization strategy. (**b**) Draining lymph node cells were cultured in the presence of E2* to evaluate the number of specific antibody-secreting cells (ASCs) by ELISpot. Statistical analysis was performed with the two-way ANOVA followed by Bonferroni’s post-hoc (**a**), or with the one-way ANOVA followed by the Tukey post-hoc test (**b**). Data represent the mean ± SD and are representative of 2 independent experiments. * *p* < 0.05, ** *p* < 0.01, *** *p* < 0.001, and **** *p* < 0.0001.

We next investigated the cellular responses using splenocytes from immunized mice pulsed with nine peptides (namely E2_1–20_, E2_231–250_, E2_261–280_, E2_271–290_, E2_281–300_, E2_321–340_, E2_341–360_, E2_351–364_, and E2_355–364_, respectively) from the E2 protein as previously identified (Figure 4a). Mice immunized with the homologous DNA strategies showed the highest number of IFN-γ-producing cells. Notably, splenocytes from the non-targeted pVAX-E2 group produced IFN-γ against all nine selected peptides (highest breath), while the group that received the DC-targeted scDEC-E2 DNA vaccine showed the highest magnitude. The lowest response was observed after homologous immunization with the recombinant E2*+ poly (I:C). The control group pVAX/poly (I:C) presented a negligible response. We also evaluated the number of antibody-secreting cells (ASCs) by B cell ELISpot. Draining lymph nodes (dLNs) from the heterologous DNA prime-E2* protein boost pVAX-E2/E2* + poly (I:C) group exhibited the highest number of ASCs (Figure 4b), while the scDEC-E2/E2* + poly (I:C) and homologous E2* + poly (I:C) groups presented similar numbers. On the other hand, homologous pVAX-E2 DNA immunization presented the lowest number of ASCs, while both the scDEC-E2 and control groups exhibited negligible responses.

Finally, analysis of the T cell profile (Figure S6) revealed that heterologous prime-boost strategies induced higher IFN-γ- or TNF-α-producing CD4^+^ and CD8^+^ T cells compared to the homologous strategies. These data led us to conclude that heterologous prime-boost immunization was the most efficient in inducing both the specific humoral and cellular immune responses.

### 2.5. Immunization with Recombinant E2* Protein in the Presence of Different Adjuvant Formulations Elicits Neutralizing Antibodies

Our previous results showed that immunization with recombinant E2* protein in homologous or heterologous prime-boost was superior to homologous DNA immunization to induce antibody responses. Therefore, we next sought to investigate the influence of poly (I:C), AddaVax, Alhydrogel, AS03, and QuilA-assisted immunization on the specific immune response.

An immunofluorescence assay showed that sera from mice immunized with E2* combined with the different adjuvants successfully recognized CHIKV-infected cells. In contrast, sera from control groups were unable to recognize the virus (Figure S7a). Analysis of E2*-specific humoral responses (Figure S7b) demonstrated that after the first dose, sera from mice that received the recombinant protein admixed with AS03 or Quil A exhibited higher antibody titers. After the boost, antibody titers increased significantly across all groups. Notably, the groups with higher IgG titers received E2* in the presence of AS03, AddaVax, or QuilA. Control groups presented negligible titers. A head-to-head comparison of the IgG subtypes (Figure S7c) showed lower IgG1/IgG2c ratios after immunization in the presence of poly (I:C), AddaVax, or AS03. Also, similar antibody affinities were observed in all immunized groups, except for the E2*+ Alhydrogel group, which presented a lower affinity (Figure S7d). In addition, sera from all groups that received the E2* protein were able to neutralize CHIKV infection (Figure S7e), but immunization in the presence of poly (I:C), AS03, or AddaVax presented a higher NT_50_. In contrast, control groups presented a negligible neutralization potential.

Furthermore, we also characterized the IFN-γ response against the previously identified immunodominant E2_231–250_ peptide (Figure S8a). Immunization with E2* + poly (I:C) induced the highest number of IFN-γ-producing cells followed by immunization with E2* + AS03. On the other hand, mice that received the protein combined with AddaVax, Alhydrogel, or QuilA presented lower levels of the IFN-γ response. In particular, the number of antibody-secreting cells was higher in the dLNs from the E2* + AS03 immunized group compared to the other adjuvants, while the lowest number was observed after immunization with E2* + Alhydrogel (Figure S8b).

Taken together, these results led us to select AS03 as the adjuvant for the following experiments based on the induction of a robust humoral response with neutralizing capacity and promising results on cellular immunity.

### 2.6. AS03 Induces Robust Responses in Heterologous DNA prime-E2* Protein Boost

**Figure 5:**
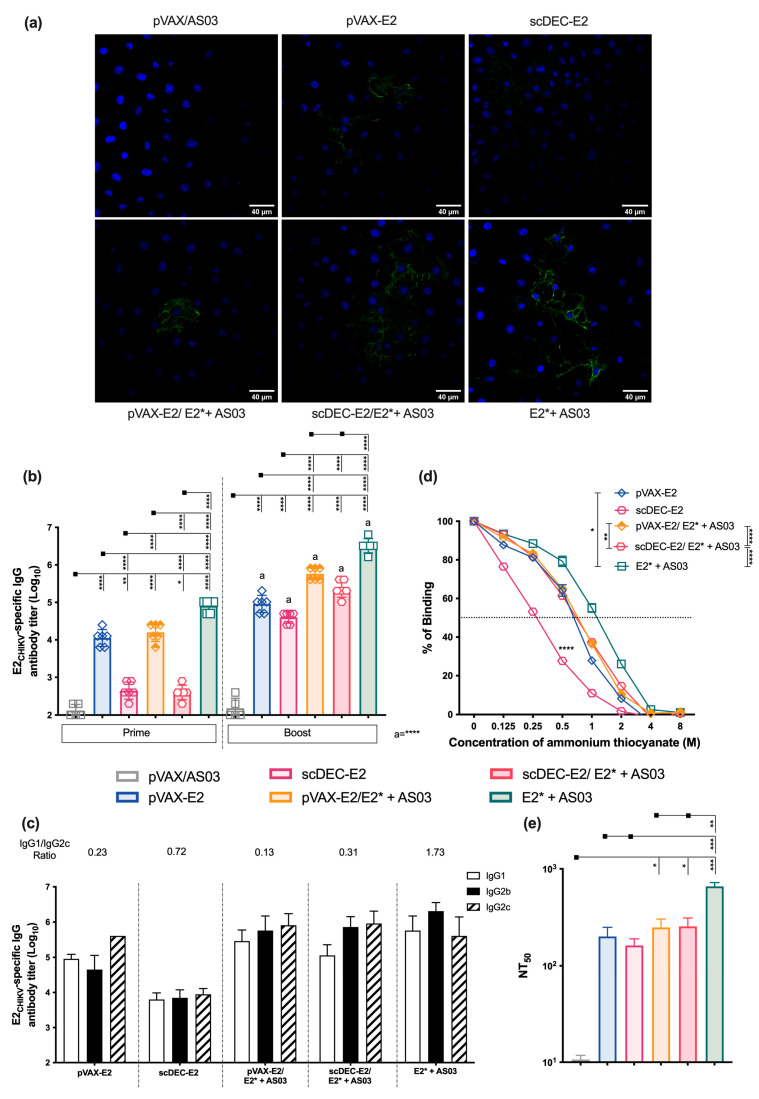
Immunization in the presence of AS03 adjuvant elicits robust humoral responses with a strong neutralizing ability. C57BL/6 mice were immunized intramuscularly twice 15 days apart with 100 μg of the non-targeted pVAX-E2 DNA vaccine or the DC-targeted scDEC-E2 DNA vaccine followed by electroporation, or with 10 μg of E2* recombinant protein + AS03 subcutaneously (immunization strategy displayed in Figure S2a). For the heterologous prime-boost, mice received one dose of a DNA vaccine (pVAX-E2 or scDEC-E2) followed by E2* recombinant protein + AS03. The control group received the empty pVAX vector and AS03. Blood samples were collected 14 days after each immunization to evaluate the antibody response. (**a**) Vero E6 cells were infected with CHIKV virus (MOI = 0.1) for 20 h, incubated with pooled sera, followed by donkey-anti mouse IgG-Alexa Fluor 488 and DAPI staining. (**b**) Total E2*-specific IgG titers. (**c**) E2*-specific IgG subclasses after the boost on a logarithm scale. (**d**) Antibody affinity from pooled sera after incubation with increasing concentrations of ammonium thiocyanate. (**e**) For PRNT, pooled sera were incubated with 100 PFU of CHIKV and the NT_50_ is displayed. Statistical analysis was performed with the one-way ANOVA followed by the Tukey post-hoc test. Data represent the mean ± SD. a- statistical significance conducted when compared to the first dose. * *p* < 0.05, ** *p* < 0.01, *** *p* < 0.001, and **** *p* < 0.0001.

We next investigated the effects of AS03 in the heterologous DNA prime-E2* protein boost. C57BL/6 mice were immunized twice as previously described (homologous and heterologous strategies). We then analyzed the specific recognition of CHIKV-infected cells with immunofluorescence (Figure 5a). Sera from all groups recognized the virus except for the control group. Sera from all experimental groups presented antigen-specific antibodies, with higher levels observed in mice that received E2* protein + AS03. After the boost, specific titers significantly increased mainly in the homologous E2* protein + AS03 and the heterologous DNA prime-E2* + AS03 boost groups (Figure 5b). Homologous and heterologous prime-boost containing DNA vaccines induced a lower IgG1/IgG2c ratio compared to homologous E2* + AS03 (Figure 5c). On the other hand, the highest antibody affinity was observed in the homologous E2* + AS03 and heterologous pVAX-E2/E2* + AS03 boost groups. As previously observed, the homologous scDEC-E2 group presented the lowest antibody affinity (Figure 5d). Furthermore, the highest neutralizing ability was detected after the homologous immunization with E2* + AS03. Slightly lower NT_50_ titers were observed in heterologous prime-boost groups. As expected, homologous DNA immunization induced the lowest NT_50_ titers (Figure 5e).

**Figure 6:**
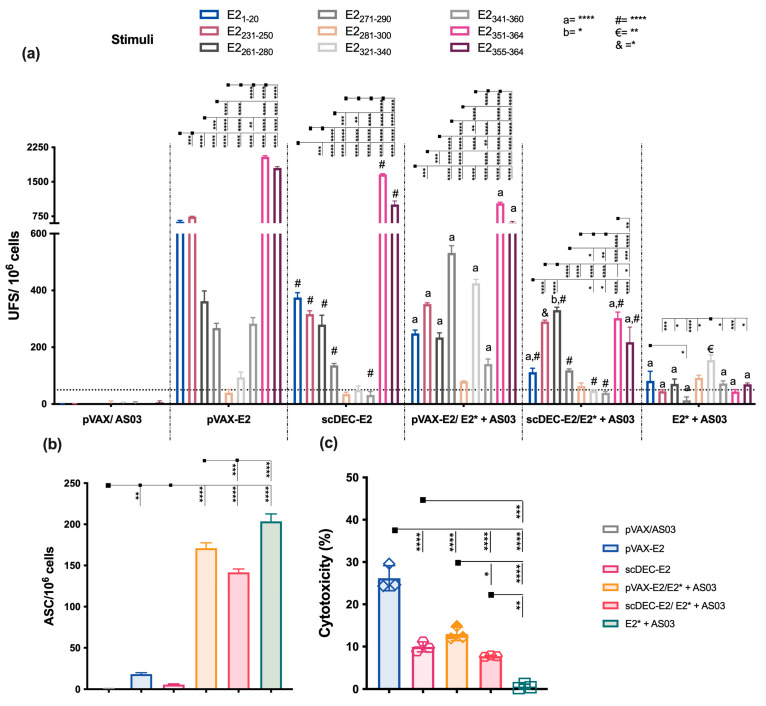
Vaccines containing E2_CHIKV_ elicit cellular immune responses with a cytotoxic profile. C57BL/6 mice (n = 6) were immunized as described in Figure 5 (immunization strategy displayed in Figure S2a). Fifteen days after the boost, mice were euthanized and spleen and draining lymph nodes were removed. (**a**) Specific IFN-γ production was examined with ELISpot against individual peptides. (a, b)) represents statistical significance between the homologous and heterologous strategies with the same vaccine used as a prime. #, €, & indicate the statistical significance between the pVAX-E2 and scDEC-E2 groups in the homologous regimen. (**b**) Draining lymph node cells were cultured in the presence of E2* to evaluate the number of specific antibody-secreting cells (ASCs) with ELISpot (**c**) In vivo cytotoxicity assay against target cells pulsed with the E2_355–364_ peptide. Statistical analysis was performed with the one-way ANOVA followed by the Tukey post-hoc test. Data represent the mean ± SD. * *p* < 0.05, ** *p* < 0.01, *** *p* < 0.001, and **** *p* < 0.0001.

Analyzes of T-cell-mediated immunity revealed that splenocytes from mice immunized with DNA in the homologous strategy presented higher numbers of IFN-γ-producing cells (Figure 6a) directly against eight of the analyzed peptides (higher breadth) and with a higher magnitude of response against the E2_1–20_, E2_231–250_, E2_351–364_ and E2_355–364_ peptides, respectively. On the other hand, heterologous prime-boost using pVAX-E2 as a prime was more efficient to induce IFN-γ-secreting cells compared to scDEC-E2. The lowest number of IFN-γ-producing cells was observed in the E2* + AS03 group. Moreover, draining lymph nodes (dLNs) from mice immunized with E2* + AS03 presented the highest number of antibody-secreting cells (ASCs) (Figure 6b), while heterologous immunization presented slightly lower ASCs.

To further characterize the T cell compartment, we performed in vivo cytotoxic T cell analysis. The highest frequency of cytotoxic CD8^+^ T cells against the target peptide-pulsed cells (E2_351–364_ peptide) was detected after homologous DNA immunization with pVAX-E2 (Figure 6c). On the other hand, homologous scDEC-E2 and heterologous pVAX-E2/E2* presented a lower and similar cytotoxic activity, while the homologous protein E2* + AS03 group did not induce significant cytotoxic T cells. Finally, homologous DNA immunization induced higher frequencies of CD4^+^ and CD8^+^ T cells producing IFN-γ and TNF-α alone or in combination that were mainly directly against E2_1–20_, E2_213–250_, and E2_351–364_ (for CD4^+^) and E2_351–364_ and E2_355–364_ (for CD8^+^) peptides, respectively (Figure S9). Immunization with E2* induced lower frequencies of cells producing cytokines.

Overall, our results suggest that the heterologous prime-boost strategy, with the pVAX-E2 DNA vaccine as the prime followed by the E2* protein + AS03 as the boost is a promising combination to elicit a broad humoral and cellular immune response.

## 3. Discussion

Over the last decades, CHIKV infection has significantly gained attention due to its ability to induce intense and debilitating arthralgia that can last for months or years, leading to a significant social and economic impact. Despite significant efforts, there are no approved prophylactic vaccines or specific treatments against this disease. Recently, a live-attenuated candidate (VLA1553) achieved a phase 3 clinical trial, showing promising results [56]. Although live-attenuated vaccines proved to be effective against some virus infections, there are some limitations for administration in immunocompromised/allergic individuals and in pregnant women. So, in recent years, extensive studies have been conducted to develop new vaccine platforms against arboviruses including zika, dengue, and chikungunya.

In the present study, we produced two DNA vaccines encoding the ectodomain of the E2_CHIKV_ envelope glycoprotein and a recombinant E2* protein produced in bacteria. To improve the immunogenicity of the DNA vaccines, we combined two different approaches: delivery of the DNA vaccine by electroporation and targeting the encoded antigen to a specialized dendritic cell subset (CD8α^+^) using a single-chain antibody to the endocytic receptor DEC205 (scDEC). Previous studies revealed promising results using DNA vaccines that targeted antigens to dendritic cells against a variety of pathogens, including HIV [50,52], influenza [57], *Yersinia pestis* [58], *P. yoelii* [59], *P. falciparum* [47], *T.cruzi* [60], *M. tuberculosis* [61], *T. gondii* [62], dengue virus [55] and cancer [63]. This platform offers attractive advantages including stability, low cost of production, and the absence of the Fc domain [51]. On the other hand, extensive studies demonstrated the ability of in vivo electroporation to increase the plasmid uptake and improve the immunogenicity and the efficacy of DNA vaccines [64]. Indeed, previous reports have shown that association of in vivo electroporation with DNA vaccines encoding E2_CHIKV_ or E1-E2-E3_CHIKV_ induced specific antibodies with a protective ability [36,37]. Due to these characteristics and a high safety profile, electroporation has been successfully tested in clinical trials including in vaccines against COVID-19 [64,65,66]. In our study, the DNA vaccine encoding the non-targeted antigen pVAX-E2 delivered using electroporation elicited superior humoral and cellular responses than the dendritic cell-targeted DNA vaccine (scDEC-E2). This is in line with a previous report, where a targeted influenza DNA vaccine was found to be less immunogenic and protective in mice compared with DNA encoding non-targeted antigens [57].

The main targets of neutralizing antibodies during CHIKV infection are the envelope glycoproteins displayed as trimers of E2/E1 heterodimers. Neutralizing antibodies induced by infection mainly target the E2 protein [23,67], and more specifically the linear epitope anti-E2EP3 previously described [68]. In fact, high neutralizing antibody titers have been associated with asymptomatic infection [69] and reduced symptoms [70]. Furthermore, human anti-E2_CHIKV_ monoclonal antibodies have been discovered to inhibit the infection in mice [23,71]. Our results showed that the homologous E2* protein and the heterologous non-target pVAX-E2 DNA vaccine prime- E2* protein boost induced high IgG titers with neutralizing capacity. On the other hand, homologous immunization with the DC-targeted scDEC-E2 DNA vaccine induced the lowest response. In humans, PRNT titers above 10 have already been shown to protect against the development of symptom development during infection [70]. Previous studies have also revealed that homologous immunization with the E2 protein in the presence of different adjuvants was able to induce higher specific antibody titers [72] and protection against challenge [31].

The cellular immune response, including IFN-γ production by T cells, also plays a critical role in controlling virus replication [73]. Moreover, the induction of robust cellular immune responses is a desirable vaccine ability, and mapping the specific sequences recognized by T CD4^+^/CD8^+^ T cells is a powerful tool to design vaccines. Here, DNA immunization in both homologous and heterologous regimens generated a large number of IFN-γ-secreting cells, as previously observed with a DNA vaccine encoding E2 [36,74] or E1 + E2 + E3 [37]. Mapping of the immunogenic epitopes revealed that high numbers of IFN-γ-secreting cells were directed to E2_1–20_ and E2_21–40_, which were also described after immunization of BALB/c mice with a peptide vaccine plus CpG ODN [75], and after CHIKV infection in mice [76]. The IILYYYELY epitope, which was present in our E2_351–364_-peptide, was predicted in silico as a possible immunogenic sequence [77], and was identified after immunization of C57BL/6 mice with a DNA vaccine encoding E2 [36].

Strategies that combine different vaccine platforms during the prime and boost phases using the same antigen are known as heterologous prime-boost (or mix-and-match), and have been used successfully against a variety of diseases, including HIV [78], seasonal influenza [79], and, more recently, COVID-19 [80]. We observed that the heterologous non-targeted pVAX-E2 DNA vaccine prime- E2* protein + AS03 boost was the best strategy to induce both robust humoral immune responses with neutralizing ability and cellular immunity. In fact, when we compared the IgG titers after two doses of homologous DNA immunization (pVAX-E2) with the heterologous DNA prime-E2*protein + AS03 boost, we observed that heterologous immunization induced a 5–6-fold change in total IgG titers and a 1.2-fold increase in the neutralizing titers, respectively. Previous work with chikungunya vaccines expressing the CHIKV envelope proteins also presented a similar profile after heterologous replicon DNA vaccine (DREP) prime MVA virus boost [81,82] or DREP-prime protein boost [83], that resulted in protection against CHIKV infection in both mice [82] and non-human primates [81]. This highlights the potential to improve immunogenicity and efficacy by combining multiple vaccine platforms.

Different adjuvant formulations can directly affect the specificity, affinity, and functional profile of the antibody response [84,85]. Alum-based adjuvants, such as Alhydrogel are the most widely used in human vaccines, albeit with a poor induction of cellular immune responses [86]. On the other hand, poly (I:C) adjuvant is a synthetic analog of double-stranded RNA that induces innate and adaptative immune responses by activation of the TLR3, MDA-5, and RIG-I receptors, and also induces DC maturation [87]. AddaVax (analogous to MF59) and AS03 adjuvants are oil-in-water emulsions that are efficient to induce robust adaptative immunity and are currently used in influenza vaccines [88]. Here, we evaluated the quality of the induced response after immunization in the presence of Alhydrogel, QuilA, poly (I:C), AddaVax, and AS03. Our data showed that E2* protein + AS03 induced the highest neutralization titers, while AddaVax and poly (I:C) displayed lower levels.

In summary, we produced different vaccines based on the E2 envelope protein of CHIKV and systematically evaluated the immune response in a preclinical setting. Collectively, our data demonstrate that the heterologous DNA prime-protein boost with pVAX-E2/E2* + AS03 induced a more robust humoral response with neutralizing ability and cellular immunity. We suggest that such a combination based on E2_CHIKV_ may be particularly valuable for designing new CHIKV vaccine candidates.

## 4. Materials and Methods

### 4.1. Design and Construction of Vaccines

The E2_CHIKV_ ectodomain consensus protein sequence (aa 1 to 364) lacking the transmembrane region (aa 365 to 422) was generated after the alignment (ClustalW, MegAlign Pro V.17.4.2, DNAStar) of 79 sequences of the Brazilian virus isolates (Genbank Accession Numbers—Table S1) and synthesized by GenScript (Piscataway, NJ, USA). For the DNA vaccines, the design included mammalian codon optimization and the addition of the *Kozak* sequence. For the non-targeted pVAX-E2 DNA vaccine, the gene also included an immunoglobulin E (IgE) leader peptide sequence at the N-terminus end, and the sequence was cloned between the *Hind*III and *Xho*I sites of the vector pVAX1 (Invitrogen, Waltham, MA, USA). The construction of the DC-targeted scDEC-E2 DNA vaccine was conducted by cloning the same E2 sequence between the *Not*I and *Xba*I sites of the pcDNA 3.1 scFv αDEC205 vector as previously described [43]. The plasmids were amplified using DH5α bacteria and purified using the Endofree Plasmid Giga Kit (Qiagen, Hilden, Germany) following the manufacturer’s instructions. Finally, the integrity of the plasmids was assessed with spectrophotometry at 260 nm and in 1% agarose gel electrophoresis. For pET21a-E2* plasmid generation, the sequence was optimized for bacteria expression and amino acid residues 1 to 28 of the E2 protein ectodomain were also removed before cloning into the *Nhe*I and *XhoI* sites of the pET21a vector.

### 4.2. E2* Protein Expression and Purification in Bacteria

E2* recombinant protein was produced in BL21 (DE3) bacteria after transformation with the pET21a-E2* plasmid as previously described [74]. Briefly, bacteria were inoculated in 1 L of Luria-Bertani (LB) medium containing ampicillin (100 μg/mL) (Sigma-Aldrich, San Luis, USA), and were grown at 37 °C, 250 rpm to an optical density (OD) of 600 nm between 0.6 and 0.8, respectively. The protein expression was then induced with 0.01 mM isopropyl-b-D-thigalactoside (IPTG, Sigma-Aldrich, San Luis, USA) for 4 h at 37 °C and 200 rpm. Bacteria were harvested by centrifugation (15 min, 4 °C and 6000× *g*), resuspended in Buffer A (Tris-HCl 100 mM, NaCl 500 mM, and glycerol 15%, pH 8), and lyzed using a high-pressure system (600 bar, 10 min, 4 °C, APLAB-10, Artepeças, São Paulo, Brazil). After lysis, the bacteria were centrifuged (40 min, 10,000× *g*, 4 °C), and the inclusion bodies were solubilized in Buffer A with the addition of 8M urea and maintained under slow and constant stirring at 4 °C overnight. For protein refolding, the supernatant was diluted (20×) in the same buffer supplemented with 20 mM 2-mercaptoethanol. The E2* recombinant protein was purified using Ni-Sepharose histidine-tagged resin (GE Healthcare, Chicago, USA) according to the manufacturer’s instructions.

### 4.3. DNA Vaccines In Vitro Expression

Six-well flat-bottom plates (Costar®, Corning, NY, USA) were seeded with 5 × 10^5^ HEK293T cells/well in DMEM (Gibco, Waltham, MA, USA) supplemented with 1% (*v*/*v*) L-glutamine (Invitrogen), 1% (*v*/*v*) penicillin/streptomycin (Invitrogen), and 10% fetal bovine serum, and cultured overnight. When the cells reached approximately 70~80% confluence, the culture medium was replaced by Opti-MEM (Gibco) supplemented with 1% Nutridome (Roche, Basel, Switzerland) without antibiotics and cultured for 1 h at 37 °C, 5% CO_2_. Cells were transfected with 5 μg of plasmid DNA (pVAX-E2 or scDEC-E2) using Lipofectamine 2000 (Invitrogen), according to the manufacturer´s instructions. Cell plates were incubated for 5 days at 37 °C in 5% CO_2_. Culture supernatants and cells were individually collected, and cell lysates were obtained using a lysis buffer (150 mM NaCl (Synth, Sao Paulo, Brazil), 50 mM Tris-HCl (Merck, Darmstadt, Germany), pH 8, and 1% Triton X-100 (Sigma-Aldrich, San Luis, MO, USA). Subsequently, the disrupted cell pellet was separated by centrifugation at 10,000× *g* for 5 min at 4 °C and resuspended in PBS. The supernatant was concentrated approximately 10 times using a 30 kDa Centriprep (Millipore, Burlington, MA, USA). The samples were evaluated using 12% SDS-PAGE gels under reducing conditions and transferred to nitrocellulose membranes. Immunoblotting was performed with specific antiserum (1:500) from mice immunized with E2* recombinant protein + poly (I:C) and horseradish-peroxidase-labeled goat anti-mouse IgG (1:2000, KPL) using the ECL detection system (Thermo Fisher, Waltham, MA, USA).

### 4.4. Mice and Immunization

Female C57BL/6 mice (seven to eight weeks old) were bred at the Centro de Desenvolvimento de Modelos Experimentais para Medicina e Biologia (CEDEME- UNIFESP). All mice were housed in a temperature- and light-cycled controlled facility at the Division of Immunology, UNIFESP. All animal experimental procedures were approved by the Institutional Animal Care and Use Committee (IACUC) (protocol number #3237110316) and were in accordance with the recommendations of the Federal Law 11.794 (2008), the Guide for the Care and Use of Laboratory Animals of the Brazilian National Council of Animal Experimentation (CONCEA), and the ARRIVE guidelines (https://arriveguidelines.org, accessed on 15 July 2020). Groups of 4–6 animals were immunized with two doses, 2 weeks apart, using 100 μg of the non-targeted pVAX-E2 or DC-targeted scDEC-E2 DNA vaccines intramuscularly in association with in vivo electroporation (8 pulses of 100V with a duration of 40 milliseconds, 1 s apart) (Electroporator, ECM 830 Generator, BTX). For recombinant protein immunization, mice received two doses with 10 μg of recombinant E2* in the presence of poly (I:C) (50 μg), AddaVax (1:1 *v*/*v*), Alhydrogel (1:1 *v*/*v*), AS03 (1:1 *v*/*v*), or QuilA (15 μg) delivered subcutaneously at the base of the tail. All adjuvants were obtained from Invivogen, San Diego, USA. The heterologous DNA-prime, protein-boost immunization strategy consisted of one dose with non-targeted (pVAX-E2) or DC-targeted (scDEC-E2) DNA vaccines followed by one dose with the recombinant E2*protein in the presence of the adjuvant (immunization strategy displayed in Figure S2a). Control groups received only the empty pVAX vector and/or the adjuvant. Blood samples were collected using submandibular vein puncture 14 days after each immunization, and mice were euthanized 15 days after the last dose.

### 4.5. Immunoblot

Approximately 1 μg of recombinant E2* protein was submitted to a 12% SDS-PAGE under reducing conditions and transferred to nitrocellulose membranes (Hybond-C extra nitrocellulose- GE Healthcare, Chicago, USA). The nitrocellulose membranes were blocked with PBS 0.05% Tween 20 (PBST), fat-free milk (5% *w*/*v*) and BSA (2.5% *w*/*v*) overnight at 4 °C. The membranes were washed 3 times with PBST and incubated with immunized mice (1:500), or human (from a convalescent CHIKV patient or a non-infected individual) (1:500) sera for 2 h at room temperature. After washing with PBST, the membranes were incubated with horseradish peroxidase-labeled goat anti-mouse IgG (1:2000, KPL) or phosphatase goat anti-human IgG (1:2000, KPL) for 2 h at room temperature. After 3 washes, the reaction was developed with chemiluminescence (ECL, GE Healthcare, Chicago, USA) or BCIP/NBT kit (Invitrogen) and analyzed with Alliance 4.7 software (Uvitec, Cambridge, UK). 

### 4.6. ELISA

To evaluate E2*-specific antibody titers [74], high-binding ELISA plates (Costar®, Corning, NY, USA)) were coated with 5 μg/mL of the recombinant protein diluted in PBS overnight at room temperature. Plates were washed with 0.02% PBST after each step, and the wells were blocked with PBST, BSA (1% *v*/*v*), and nonfat milk (5% *v*/*v*) for 2 h. Sera from immunized mice were serially diluted in 100 μL of block solution and incubated for 2 h. After that, plates were incubated for 2 h with goat horseradish peroxidase-labeled anti-mouse IgG (1:10,000, KPL). Finally, the reaction was developed by adding 1 mg/mL of o-phenylenediamine (OPD, Sigma-Aldrich, San Luis, USA) diluted in phosphate–citrate buffer, pH 5, containing 0.03% (*v*/*v*) hydrogen peroxide. The reaction was stopped by adding 50 μL of a 4 N H_2_SO_4_ solution, and plates were read at OD_492nm_ with an ELISA reader (EnSpire Multimode Plate Reader; PerkinElmer, Waltham, MA, USA). Antibody titers were determined by the highest dilution between OD_492nm_ of 0.1–0.2. For antibody detection from the different IgG subclasses, the ELISA assay was performed under the same conditions described above, except for the secondary antibodies specific for mouse IgG1, IgG2b, and IgG2c (1:4000, Southern Biotech, Birmingham, AL, USA), respectively. For antibody affinity evaluation, we performed an ELISA with an extra step that included the chaotropic agent ammonium thiocyanate after sample incubation. The agent was added to the wells at concentrations between 0 and 8M. After 15 min of incubation, plates were washed several times with PBST and incubated with the secondary anti-mouse IgG antibody. The concentration of the chaotropic agent to dissociate 50% of the antibodies was determined using the formula:

$$\frac{{\mathrm{OD}}_{492\mathrm{nm}}\;\mathrm{in}\;\mathrm{the}\;\mathrm{presence}\;\mathrm{of}\;\mathrm{ammonium}\;\mathrm{thiocyanate}\;\times 100}{{\mathrm{OD}}_{492\mathrm{nm}}\;\mathrm{in}\;\mathrm{the}\;\mathrm{absence}\;\mathrm{of}\;\mathrm{ammonium}\;\mathrm{thiocyanate}}$$

### 4.7. Plaque Reduction Neutralization Assay (PRNT)

A Brazilian isolate of the CHIKV ECSA strain (Genbank: KP164569) was propagated in Vero E6 cells (ATCC CRL-1586) in MEM medium (Gibco, Waltham, MA, USA) supplemented with 10% fetal bovine serum (FBS, Gibco) and 1% (*v*/*v*) of penicillin/streptomycin (Gibco, Waltham, MA, USA)-M10) for 48 h. Then, the supernatant of the infected cells was collected, harvested, and titrated as previously described [89]. For the neutralization assay, 1 × 10^5^ cells were seeded per well in M10 and incubated overnight at 37 °C and 5% CO_2_. One day later, inactivated sera samples (56 °C for 30 min) from pooled immunized mice or from the CHIKV convalescent patient were serially diluted (1:3) in supplemented MEM containing 2% FBS, 1% penicillin/streptomycin (Gibco, Waltham, MA, USA), and 0.05% Amphotericin B (Fungizone, Gibco). Duplicate samples were mixed (1:1) with 100 PFU/well of CHIKV and incubated for 1 h at 37 °C and 5% CO_2_. Next, the mixture was added to the Vero E6 monolayer and incubated for another hour under the same conditions. The cells were then overlayed with complete MEM containing 1.6% carboxymethylcellulose (CMC, Sigma-Aldrich, San Luis, USA) and 0.05% Amphotericin B (Fungizone, Gibco, Waltham, MA, USA) and were incubated for 72 h at 37 °C and 5% CO_2_. The media was removed, and the cells were then fixed with 4% paraformaldehyde solution (Sigma-Aldrich, San Luis, USA) and stained with crystal violet 0.2% (Sigma-Aldrich, San Luis, USA). The percentage of plaque reduction was measured compared to a positive control (Vero E6 cells in the absence of sera).

### 4.8. Immunofluorescence

Fifty thousand Vero E6 cells were seeded on the top of 13 mm circular cover slips in a 24-well plate in M10 media and incubated overnight at 37 °C and 5% CO_2_. Therefore, cells were infected with CHIKV (MOI 0.1), and incubated for 20 h under the same conditions. The medium was removed, and the cells were fixed with 4% paraformaldehyde solution for 30 min. After 3 washes with PBS (5 min/wash), the cover slips were incubated with pooled mouse sera (1:500) for 1 h. After an additional wash with PBS, the cover slips were incubated with donkey anti-mouse IgG conjugated with Alexa 488 (1:500, Invitrogen, Waltham, USA) for 30 min. After another wash with PBS, cover slips were incubated with DAPI (1:1000, Invitrogen, Waltham, USA). For actin staining, the samples were stained with phalloidin–Texas Red (Invitrogen) according to the manufacturer’s instructions and previous work [90]. Finally, the stained cells were washed 3 times with PBS and mounted with the anti-quenching Fluoromount-GTM mounting medium (ThermoFisher, Waltham, MA, USA) on glass slides. Images were obtained using a Leica-SP8 confocal microscope. Z images were acquired with a 0.15 μm spacing between the sections, and all images were treated with the open-source free software ImageJ version 1.46r.

### 4.9. Spleen and Lymph Node Cell Suspension

Fifteen days after the last dose, the mice were euthanized, and their spleen and draining lymph nodes (inguinal and popliteal) were aseptically removed. Cell suspensions were treated with red blood cell lysis buffer (ammonium chloride potassium (ACK)). Cells were then washed and resuspended in RPMI 1640 media supplemented with 10% FBS, 2 mM L-glutamine, 1 mM sodium pyruvate, 1% *v*/*v* non-essential amino acids, 40 μg/mL gentamicin, and 5 × 10^−5^ M 2-mercaptoethanol (all from Gibco). Viability was evaluated with 0.2% Trypan solution, and cell concentrations were determined with a cell counter (Countess^TM^ Automated Cell Counter, Invitrogen) and adjusted in the culture medium.

### 4.10. Peptide Library

A library of 36 peptides (20-mer, with 12 amino acids overlapping (Table S2), comprising the E2_CHIKV_ protein consensus sequence (amino acids 1–364) was synthesized by GenScript, Inc. Piscataway, NJ, USA. The peptides purity (greater than 75%) was determined using high-performance liquid chromatography. The peptides were then resuspended in dimethyl sulfoxide (DMSO) and stored at −20 °C.

### 4.11. T Cell ELISpot Assay

The CHIKV-specific T cell response after in vitro stimulation with individual peptides from the E2 envelope protein was assessed using the ELISpot assay. The ELISpot assay was performed using the IFN-γ ELISpot Ready-SET-Go! Kit (eBiociences, San Diego, CA, USA) according to the manufacturer’s instructions. Three hundred thousand splenocytes were added per well and stimulated with pooled peptides generated after a matrix strategy using the DeconvoluteThis! Software (v. 2.0) as previously described [91] (Table S3), with individual peptides from the E2_CHIKV_ envelope protein (10 μg/mL) or R10 media (negative control) for 18h at 37 °C and 5% CO_2_. Spots were counted using an AID ELISpot reader system (Autoimmun Diagnostika GMbH, Straberg, Germany).

### 4.12. B Cell ELISpot Assay

The frequency of antigen-specific antibody secreting cells (ASCs) was evaluated by ELISpot. Briefly, plates were coated with the E2* recombinant protein (5 μg/mL) and incubated overnight. The plates were then blocked using R10 media for 2 h and 1 × 10^6^ lymph node cells per well were then added and incubated for 18 h at 37 °C, with 5% CO_2_. Next, plates were washed with PBS and incubated with horseradish peroxidase goat anti-mouse IgG (1:1000, KPL) for 2 h. After two additional washes, the reaction was developed with 3-amino-9-ethylcarbazole (AEC; BD Biosciences, Franklin Lakes, NJ, USA), and the spots were counted using an AID ELISpot reader system (Autoimmun Diagnostika GMbH, Straberg, Germany).

### 4.13. Polyfuncional E2_CHIKV_-Specific T Cell Response by Flow Cytometry

To evaluate E2_CHIKV_-specific CD4^+^ and CD8^+^ T cell cytokine production, splenocytes were isolated and cultured in R10 overnight in 96-well round bottom plates (1 × 10^6^ cells/well, in triplicate) at 37 °C and 5% CO_2_ with media only, or with E2_CHIKV_ individual peptides (10 μg/mL) in the presence of anti-CD28 (2 μg/mL, BD Pharmigen, Franklin Lakes, NJ, USA) and Brefeldin A GolgiPlug® (BD Pharmigen, New Jersey, USA) for 12 h. The cells were then washed with Macs buffer and surface stained for 30 min on ice with anti-CD3-APCCy7 (clone 145–2C11), anti-CD4-PerCP (clone RM4-5), and anti-CD8-Pacific Blue (clone 53–6.7). After staining, cells were washed, fixed, and permeabilized using a Cytofix/Cytoperm® kit (BD Pharmigen), following the manufacturer’s instructions. The samples were washed with the Perm/Wash buffer (BD Pharmigen) and stained intracellularly with IFN-γ-APC (clone XMG1.2) and TNF-α-PeCy7 (clone MP6-XT22) for 30 min on ice (all from BD Pharmigen). Subsequently, cells were washed twice and resuspended in the Macs buffer. The samples were acquired on a FACSCanto II flow cytometer (BD Biosciences) and then analyzed using FlowJo software (version 10.4, Tree Star, Elmwood Park, NJ, USA). To analyze the polyfunctional profile of T cells, we used the Boolean gating platform (FlowJo 10.2) to create combinations of the cytokines. The frequency of cytokine-producing cells were calculated by subtracting the values from the non-stimulated cells. Unstained and all single-color controls were processed to allow for proper compensation.

### 4.14. In Vivo Cytotoxicity Assay

Splenocytes from C57BL/6 naive mice were either pulsed, or not, for 1 h at 37 °C with the E2 peptide (10 μg/mL) comprising amino acids 351–364. Subsequently, the splenocyte population pulsed with the peptide was stained with carboxyfluorescein diacetate succinimidyl ester (CFSE, Molecular Probes) at a final concentration of 5 μM (CFSE^high^), and the non-pulsed population with 0.625 μM of CFSE (CFSElow). Next, a mixture of both populations of equal parts was transferred intravenously (2 × 10^7^ cells/each) into immunized mice. Then, 20 h after transfer, spleen cells were isolated, and the populations were analyzed by flow cytometry in a FACSCantoII flow cytometer (BD Biociences). One hundred thousand events were acquired inside the CFSE^low^ cell population (representative gating strategy shown in Figure S10). The percentage of specific lysis was determined using the formula as previously described [92].

### 4.15. Data Analysis

Statistical analysis was performed using the one-way ANOVA statistical test followed by the Tukey’s honestly significant difference (HSD) post-hoc test, or a two-way ANOVA followed by Bonferroni´s post-hoc test. For NT_50_ analysis, a nonlinear regression was calculated. Statistical analysis and graphical representation were conducted using GraphPad Prism (Boston, MA, USA) version 9.4 software.

## Data Availability

The data that support the findings of this study are available from the corresponding authors upon reasonable request.

## Supplementary Materials

The following supporting information can be downloaded at: https://www.mdpi.com/article/10.3390/ijms241310517/s1.
