# Supplementary material for: Heterologous DNA Prime- Subunit Protein Boost with Chikungunya Virus E2 Induces Neutralizing Antibodies and Cellular-Mediated Immunity
Source: Int J Mol Sci. 2023 Jun 23;24(13):10517. doi: 10.3390/ijms241310517 (PMC10342165; doi:10.3390/ijms241310517)
Supplement: Supplementary file 1 [file ijms-24-10517-s001.zip › ijms-2431952-supplementary.pdf]

Supplementary Material

Table S1. Brazilian Chikungunya isolates

	CHIKV Isolate	Accession Number Genbank
1	BHI3734/H804698	KP164568.1
2	BHI3741/H804705	KP164569.1
3	BHI3745/H804709	KP164570.1
4	RJ-IB1	KY124328.1
5	RJ-IB5	KY124329.1
6	Bahia08	KU940225.1
7	BR33	KX228391.1
8	C302F/2016/BR	KY055011.1
9	CHIKV/ Homo_sapiens/Brazil/ 2016/ 35AP	KY704955.1
10	CHIKV/ Homo_sapiens/Brazil/ 2016/ 243	KY704953.1
11	CHIKV/ Homo_sapiens/Brazil/ 2016/ 241	KY704952.1
12	CHIKV/ Homo_sapiens/Brazil/ 2016/ 197	KY704951.1
13	CHIKV/ Homo_sapiens/Brazil/ 2016/ 195	KY704950.1
14	CHIKV/ Homo_sapiens/Brazil/ 2016/ 194	KY704949.1
15	CHIKV/ Homo_sapiens/Brazil/ 2016/ 190	KY704947.1
16	CHIKV/ Homo_sapiens/Brazil/ 2016/ 178	KY704944.1
17	CHIKV/ Homo_sapiens/Brazil/ 2016/ 175	KY704943.1
18	CHIKV/ Homo_sapiens/Brazil/ 2016/ 172	KY704942.1
19	CHIKV/ Homo_sapiens/Brazil/ 2016/ 167	KY704940.1
20	CHIKV/ Homo_sapiens/Brazil/ 2016/ 166	KY704939.1
21	CHIKV/ Homo_sapiens/Brazil/ 2016/ 164	KY704938.1
22	CHIKV/ Homo_sapiens/Brazil/ 2016/ 17AL	KY704935.1
23	CHIKV/ Homo_sapiens/Brazil/ 2016/ 14	KY704934.1
24	CHIKV/Homo_sapiens/Brazil/2016/19	KY704936.1
25	CHIKV/ Homo_sapiens/Brazil/ 2016/ 19AP	KY704954.1
26	CHIKV/ Homo_sapiens/Brazil/ 2016/ 188	KY704946.1
27	TR206/H804187	KP164572.1*
28	AMA2798/H804298	KP164567.1*
29	PER160/H803609	KP164571.1*
30	RJ/CHIKV/2015	KU355832.1*
31	MT01	MH823663.1
32	MT02	MH823664.1
33	MT03	MH823665.1
34	MT04	MH823666.1
35	MT05	MH823667.1
36	MT06	MH823668.1
37	CHIKV/Human/Brazil/2015/AMA290	MK121891.1

38	CHIKV/Human/Brazil/2015/AMA291	MK121892.1
39	CHIKV/Human/Brazil/2015/AMA292	MK121893.1
40	CHIKV/Human/Brazil/2016/AMA293	MK121894.1
41	CHIKV/Human/Brazil/2017/AMA74	MK121895.1
42	CHIKV/Human/Brazil/2017/AMA346	MK121896.1
43	CHIKV/Human/Brazil/2017/AMA352	MK121898.1
44	CHIKV/Human/Brazil/2017/AMA354	MK121899.1
45	CHIKV/Human/Brazil/2017/AMA362	MK121900.1
46	CHIKV/Human/Brazil/2017/AMA364	MK121901.1
47	CHIKV/Human/Brazil/2017/AMA366	MK121902.1
48	CHIKV/Human/Brazil/2017/AMA368	MK121903.1
49	CHIKV/Human/Brazil/2017/AMA369	MK121904.1
50	CHIKV/Human/Brazil/2017/AMA379	MK121906.1
51	CHIKV/Human/Brazil/2017/AMA381	MK121907.1
52	BRZ-38_Brazil-RJ_2016/03/29	MG649970.1
53	BRZ-18_Brazil-RJ_2016/08/22	MG649971.1
54	BRZ-10_Brazil-RJ_2016/05/05	MG649972.1
55	BRZ-37_Brazil-RJ_2016/12/12	MG649973.1
56	BRZ-3_Brazil-RJ_2016/04/08	MG649974.1
57	BRZ-4_Brazil-RJ_2016/07/27	MG649975.1
58	BRZ-20_Brazil-RJ_2016/03/29	MG649976.1
59	BRZ-1_Brazil-RJ_2016/07/28	MG649977.1
60	BRZ-14_Brazil-RJ_2017/03/16	MG649978.1
61	BRZ-8_Brazil-RJ_2017/03	MG649979.1
62	BRZ-7_Brazil-RJ_2016/04/27	MG649980.1
63	BRZ-2_Brazil-RJ_2016/07/28	MG649981.1
64	BRZ-12_Brazil-RJ_2017/03/24	MG649982.1
65	BRZ-6_Brazil-RJ_2016/03/28	MG649983.1
66	CHK_5_Brazil-RJ_2015	MG649984.1
67	CHK_7_Brazil-RJ_2015	MG649985.1
68	BeAr849404	MK518395.1
69	10C	MH000706.1
70	7C	MH000705.1
71	6C	MH000704.1
72	5C	MH000703.1
73	4C	MH000702.1
74	2C	MH000700.1
75	BeAr843521	MT526900.1
76	BeAr843523	MT526901.1
77	BeAr843528	MT526902.1
78	BeAr843529	MT526903.1
79	BeAr843544	MT526904.1

* Asian genotype

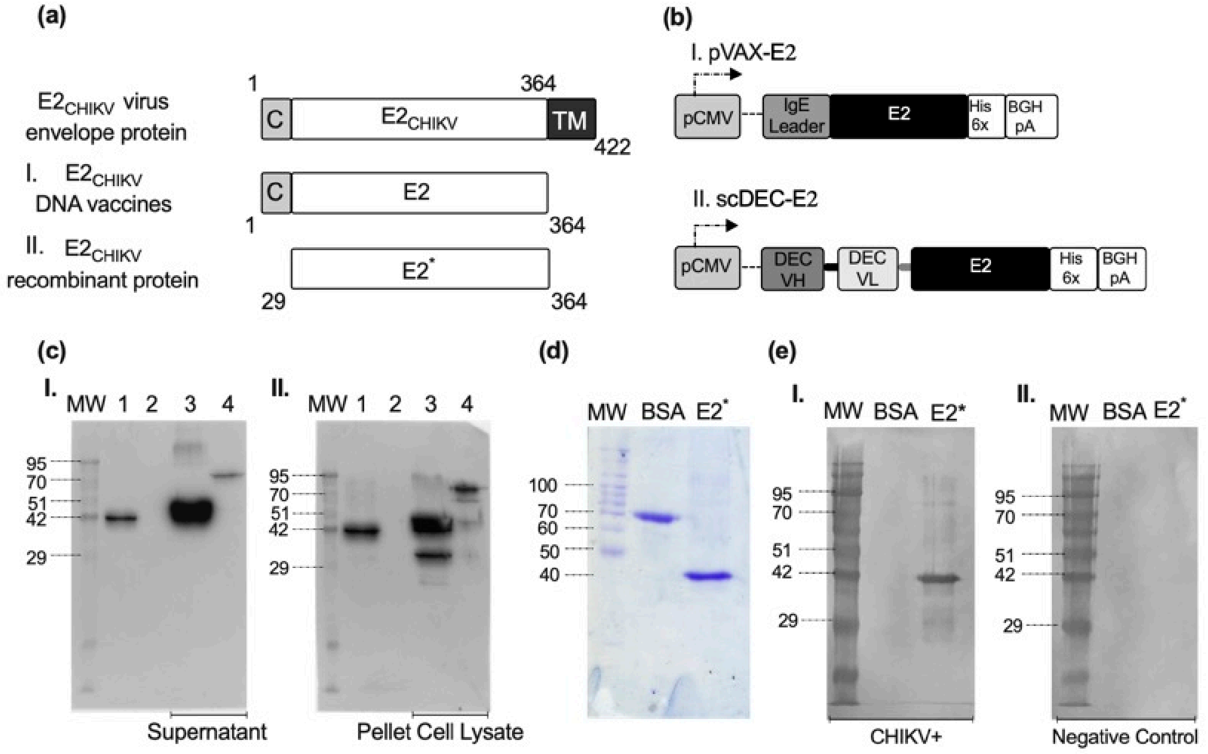

Figure S1. Design and characterization of DNA vaccines and recombinant E2*protein. (**a**) E2CHIKV consensus was generated after alignment of Brazilian CHIKV isolate sequences and optimization for (I) eukaryotic cell expression without the transmembrane portion and (II) prokaryotic cell expression without the transmembrane and N-terminus region. C. cysteine N-terminus (aa 1-24). (**b**) Schematic representation of DNA vaccines: (I) pVAX-E2 and (II) scDEC-E2. (**c**) Immunoblotting analysis, under reducing conditions, of antigen expression after HEK293T transient transfection using serum from mice immunized with E2* + poly (I:C). I. Supernatant; II. Cell lysate. 1. E2*, 2. BSA, 3. pVAX-E2, 4. scDEC-E2. (**d**) SDS-PAGE analysis of the purified E2* recombinant protein. (**e**) Immunoblot of E2* using serum from (I) a convalescent CHIKV$^+$ or (II) a non-infected individual. MW: molecular weight. BSA: bovine serum albumin; sc: single chain; E2* recombinant E2 protein lacking transmembrane and N-terminus region.

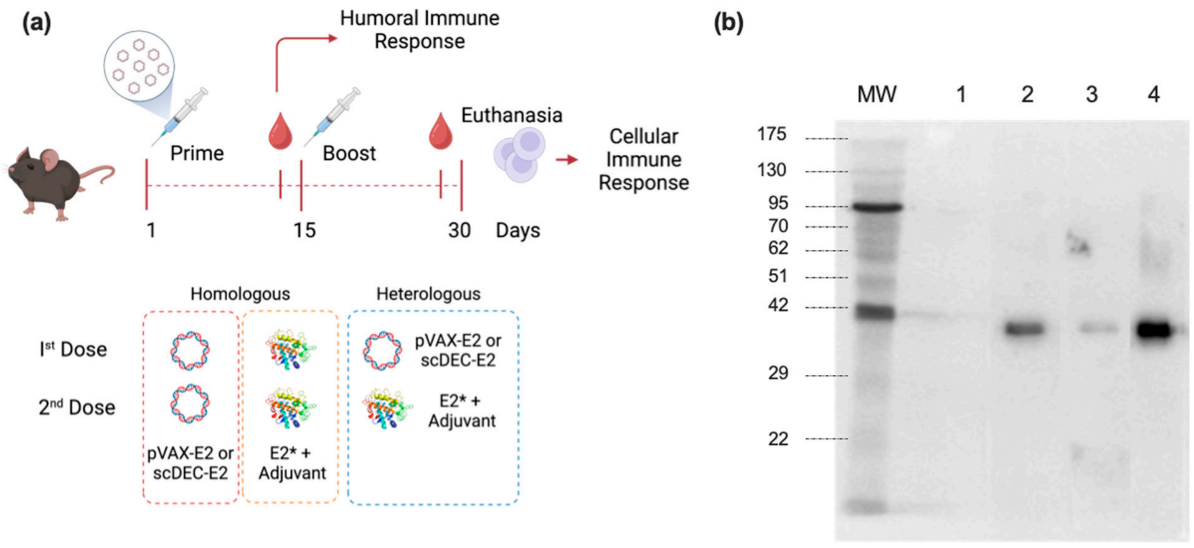

Figure S2. Immunization with E2CHIKV-based vaccines induces specific antibodies. (**a**) Immunization strategy. C57BL/6 mice were immunized intramuscularly twice with 100 µg of the non-targeted pVAX-E2 DNA vaccine or a DC-targeted scDEC-E2 DNA vaccine followed by electroporation, or with 10 µg of E2* recombinant protein + adjuvant subcutaneously. Control group received the empty pVAX vector and adjuvant. Blood samples were collected 14 days after each immunization to evaluate humoral immune response and mice were euthanized 15 days after the last dose. (**b**) Recognition of E2* under reducing conditions by pooled sera from immunized mice after boost. 1. pVAX/poly (I:C); 2. pVAX-E2; 3. scDEC-E2; 4. E2*+poly (I:C).

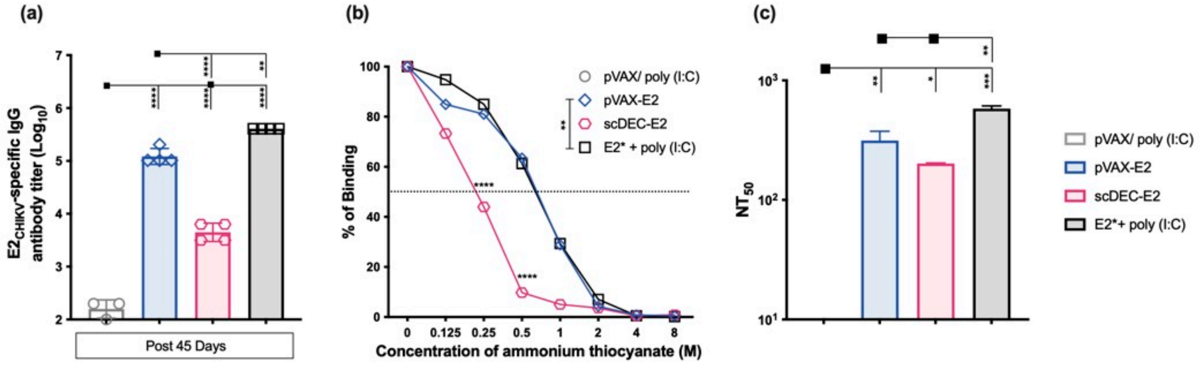

Figure S3. Specific humoral immune response is sustained 45 days after immunization. C57BL/6 mice were immunized as described in Figure 1. (**a**) Total E2*-specific IgG titers. (**b**) Antibody affinity from pooled mouse sera after incubation with increasing concentrations of ammonium thiocyanate. (**c**) For PRNT, pooled sera were incubated with 100 PFU of CHIKV and NT_{50} is displayed. Statistical analysis was performed by One-way ANOVA followed by Tukey post-hoc test. Data represent mean ± SD. *p<0.05, **p<0.01, ***p<0.001 and ****p<0.0001.

Table S2. Peptide library (20-mer overlapping 12) based on $E2_{CHIKV}$ consensus sequence.

Number	Peptide	Amino acid sequence
1	$E2_{1-20}$	STKDNFNVYKATRPYLAHCP
2	$E2_{11-30}$	ATRPYLAHCPDCGEGHSCHS
3	$E2_{21-40}$	DCGEGHSCHSPVALERIRNE
4	$E2_{31-50}$	PVALERIRNEATDGTLKIQV
5	$E2_{41-60}$	ATDGTLKIQVSLQIGIKTDD
6	$E2_{51-70}$	SLQIGIKTDDSHDWTKLRYM
7	$E2_{61-80}$	SHDWTKLRYMDNHTPADAER
8	$E2_{71-90}$	DNHTPADAERAGLFVRTSAP
9	$E2_{81-100}$	AGLFVRTSAPCTITGTMGHF
10	$E2_{91-110}$	CTITGTMGHFILTRCPKGET
11	$E2_{101-120}$	ILTRCPKGETLTVGFTDSRK
12	$E2_{111-130}$	LTVGFTDSRKISHSCTHPFH
13	$E2_{121-140}$	ISHSCTHPFHHDPPVIGREK
14	$E2_{131-150}$	HDPPVIGREKFHSRPQHGKE
15	$E2_{141-160}$	FHSRPQHGKELPCSTYVQST
16	$E2_{151-170}$	LPCSTYVQSTAATTEEIEVH
17	$E2_{161-180}$	AATTEEIEVHMPPDTPDRTL
18	$E2_{171-190}$	MPPDTPDRTLMSQQSGNVKI
19	$E2_{181-200}$	MSQQSGNVKITVNGQTVRYK
20	$E2_{191-210}$	TVNGQTVRYKCNCGGSNEGL
21	$E2_{201-220}$	CNCGGSNEGLITTDKVINNC
22	$E2_{211-230}$	ITTDKVINNCKVDQCHAAVT
23	$E2_{221-240}$	KVDQCHAAVTNHKKWQYNSP
24	$E2_{231-250}$	NHKKWQYNSPLVPRNAELGD
25	$E2_{241-260}$	LVPRNAELGDRKGKIHIPFP
26	$E2_{251-270}$	RKGKIHIPFPLANVTCRVPK
27	$E2_{261-280}$	LANVTCRVPKARNPTVTYGK
28	$E2_{271-290}$	ARNPTVTYGKNQVIMLLYPD
29	$E2_{281-300}$	NQVIMLLYPDHPTLLSYRNM
30	$E2_{291-310}$	HPTLLSYRNMGEEPNYQEEW
31	$E2_{301-320}$	GEEPNYQEEWVTHKKEVVLT
32	$E2_{311-330}$	VTHKKEVVLTVPTEGLEVTW
33	$E2_{321-340}$	VPTEGLEVTWGNNEPYKYWP
34	$E2_{331-350}$	GNNEPYKYWPQLSTNGTAHG
35	$E2_{341-360}$	QLSTNGTAHGHPHEIILYYY
36	$E2_{351-364}$	HPHEIILYYYELYP

Table S3. E2CHIKV Peptide Matrix

**Pool**	**Peptides**								
Pool 1	1	2	3	4	5	6	7	8	9
Pool 2	10	11	12	13	14	15	16	17	18
Pool 3	19	20	21	22	23	24	25	26	27
Pool 4	28	29	30	31	32	33	34	35	36
Pool 5	1	2	3	10	11	19	20	28	29
Pool 6	4	5	12	13	21	22	30	31	32
Pool 7	6	7	14	14	23	24	33	34	35
Pool 8	8	9	16	17	18	25	26	27	36

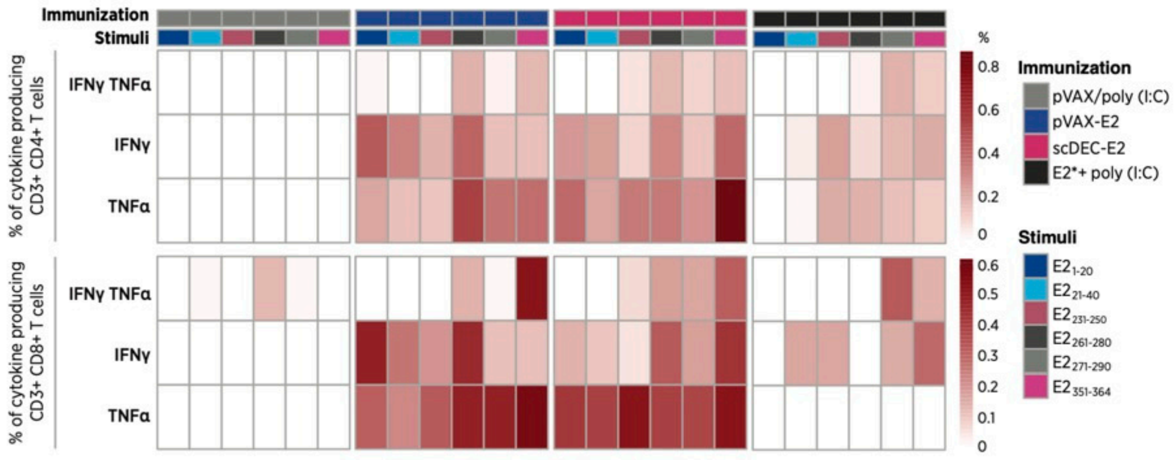

Figure S4. Immunization with vaccines representing E2CHIKV induces cytokine-producing CD4+ and CD8+ T cells. C57BL/6 mice were immunized as described in Figure 1. Fifteen days after boost, splenocytes were cultured for 12 hours in the presence of specific E2-peptides. Cells were then stained with anti-CD3, -CD4, -CD8, -IFN-γ and -TNF-α and analyzed by flow cytometry. Heatmap showing the frequency of cytokine-producing T cells calculated by subtracting the values from non-stimulated cells. Dark red represents higher production of the cytokines. Boolean combinations were created using the FlowJo platform. Data are representative of 3 independent experiments.

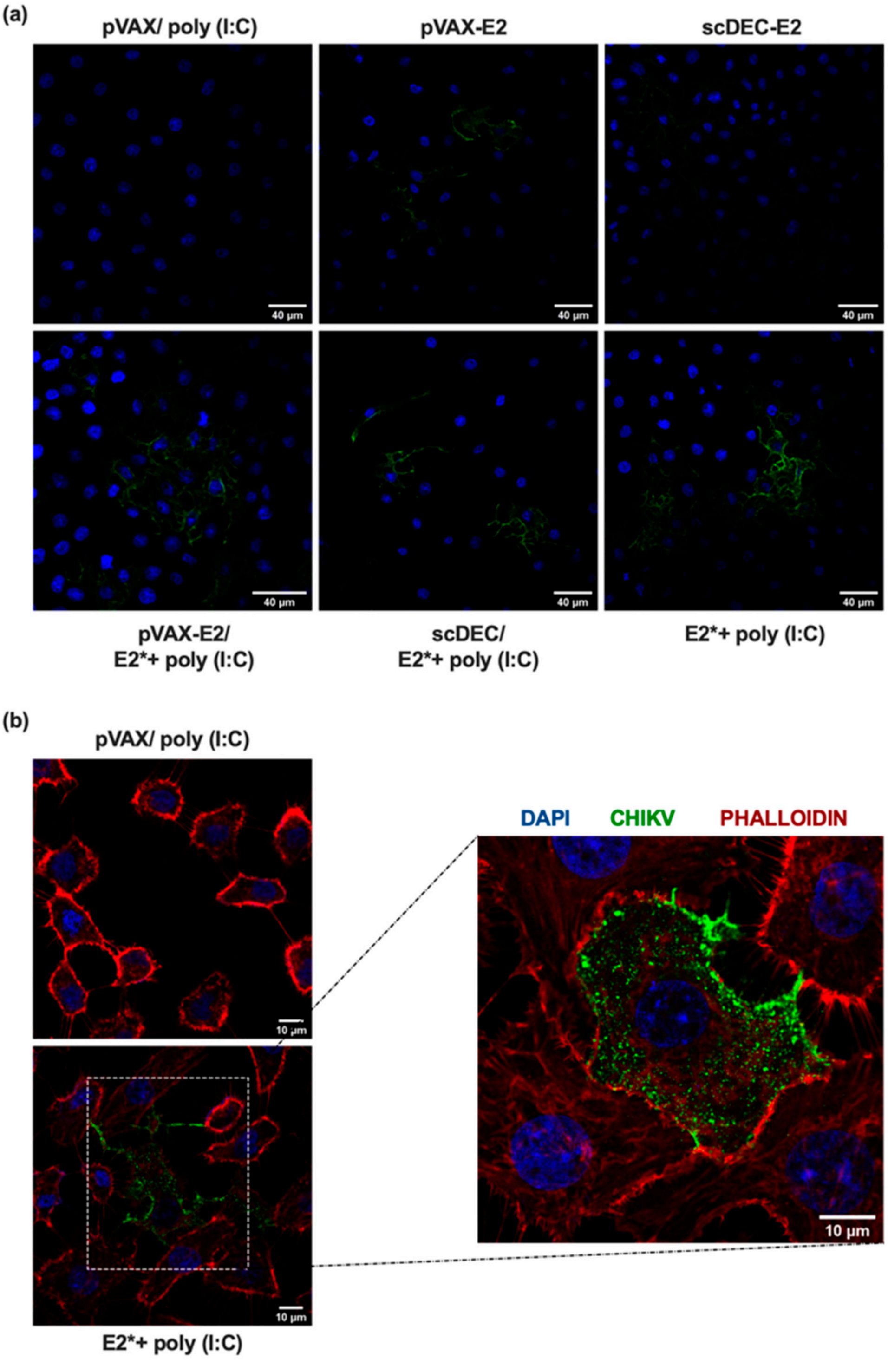

Figure S5. Sera from immunized mice specifically recognized CHIKV infected cells *in vitro*. Vero E6 cells were infected with CHIKV (MOI=0.1) for 20 hours, incubated with pooled sera from immunized mice followed by staining with (**a**) donkey-anti mouse IgG-Alexa Fluor 488 and DAPI or (**b**) donkey-anti mouse IgG-Alexa Fluor 488, DAPI and phalloidin-Texas red.

Video S1. Sera from immunized mice specifically recognizes CHIKV in infected cells. Vero E6 cells were infected with CHIKV (MOI=0.1) and incubated for 20 hours with sera from E2* + poly (I:C) immunized mice followed by staining with donkey anti-mouse IgG labeled with Alexa 488. Then, cells were labeled with DAPI and phalloidin. The images were acquired in a confocal microscope Leica-SP8. The Z-images were acquired with 0.15 μm spacing between the sections.

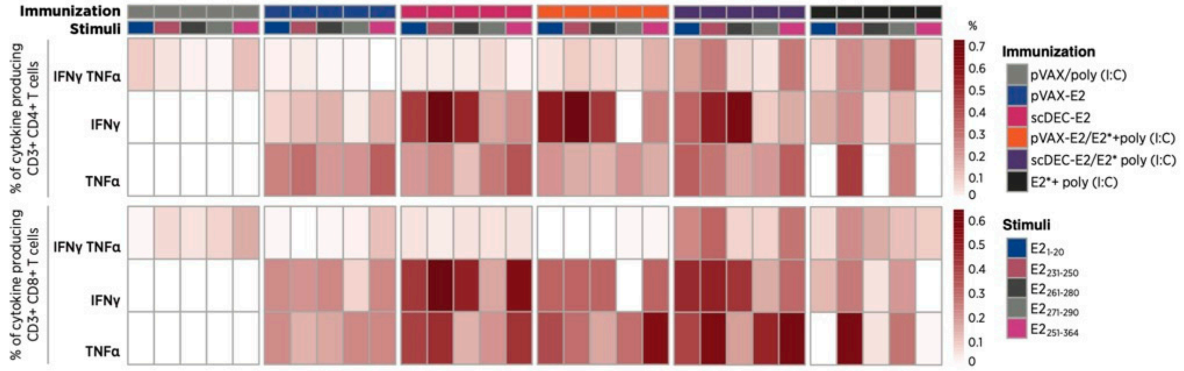

Figure S6. Heterologous prime-boost immunization induces cytokine-producing CD4$^+$ and CD8$^+$ T cells. C57BL/6 mice were immunized intramuscularly twice 15 days apart with 100 μg of the non-targeted pVAX-E2 DNA vaccine or a DC-targeted scDEC-E2 DNA vaccine followed by electroporation, or with 10 μg of E2* recombinant protein + poly (I:C) subcutaneously. For heterologous prime-boost, mice received one dose of DNA vaccine (pVAX-E2 or scDEC-E2) followed by the E2* recombinant protein + poly (I:C). The control group received the empty pVAX vector and poly (I:C). Fifteen days after boost, splenocytes were cultured for 12 hours in presence of specific E2-peptides. Cells were then stained with anti-CD3, -CD4, -CD8, -IFN-γ and -TNF-α and analyzed by flow cytometry. Heatmap showing the frequency of cytokine-producing T cells calculated by subtracting the values from non-stimulated cells. Dark red represents higher production of the cytokines. Boolean combinations were created using the FlowJo platform. Data are representative from 2 independent experiments.

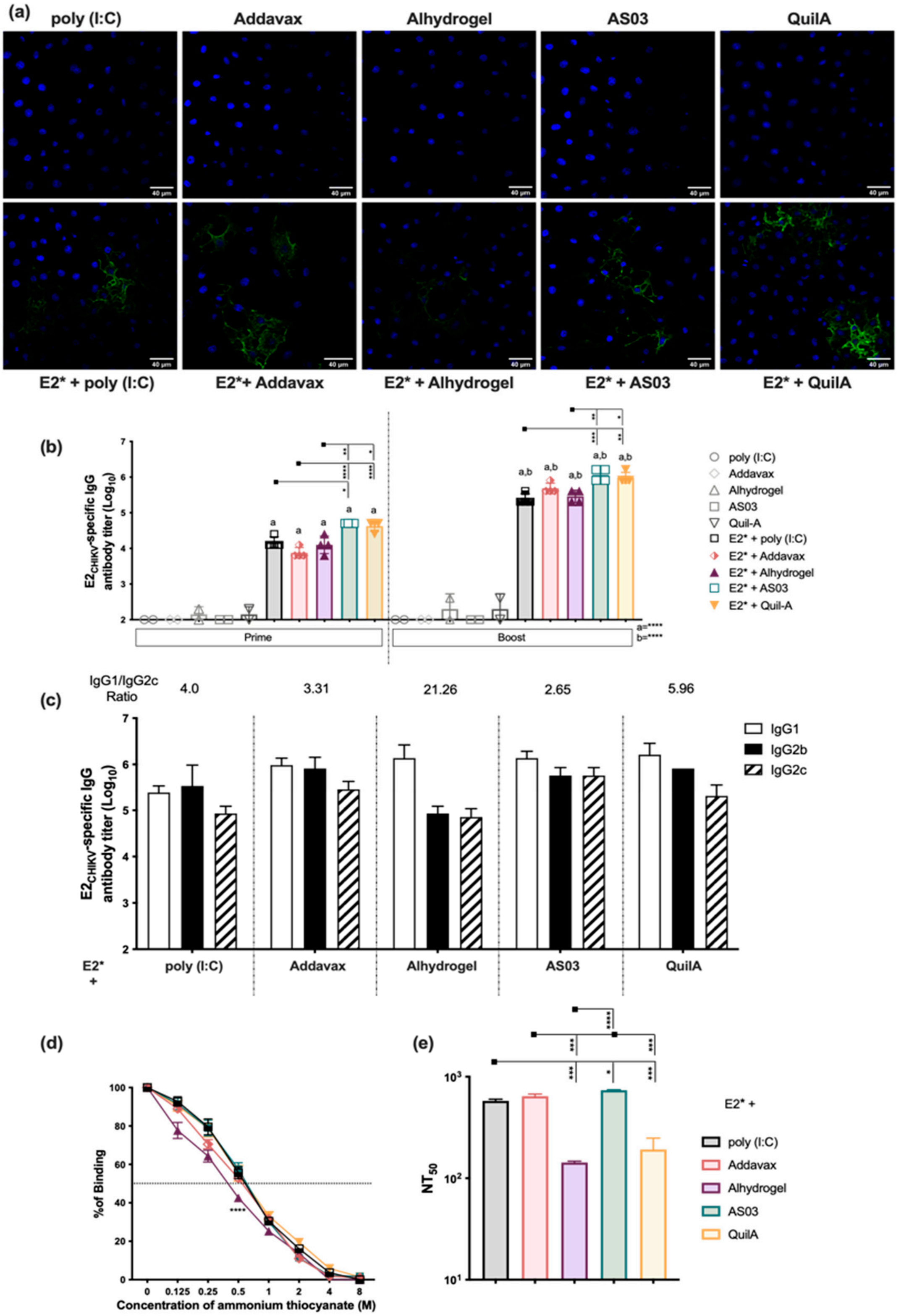

Figure S7. Different adjuvants modulate the humoral immune response induced by E2* immunization. C57BL/6 mice received 2 doses subcutaneously with 10 μg of E2*

protein in the presence of poly (I:C), Addavax, Alhydrogel, AS03 or QuilA. Blood samples were collected 14 days after each immunization to evaluate humoral immune response. (**a**) Vero E6 cells were infected with CHIKV (MOI=0.1) for 20 hours and incubated with pooled sera from immunized mice followed by staining with donkey-anti mouse IgG-Alexa Fluor 488 and DAPI. (**b**) Total E2*-specific IgG titers. a- statistical analysis in comparison to respective control group. b-statistical analysis in comparison to first dose. (**c**) E2*-specific IgG subclasses after boost. (**d**) Antibody affinity from pooled mice sera after incubation with increasing concentrations of ammonium thiocyanate. (**e**) For PRNT, pooled sera were incubated with 100 PFU of CHIKV and NT_{50} is displayed. Statistical analysis was performed by One-way ANOVA followed by Tukey post-hoc test. Data represent mean ± SD. *p<0.05, **p<0.01, ***p<0.001 and ****p<0.0001.

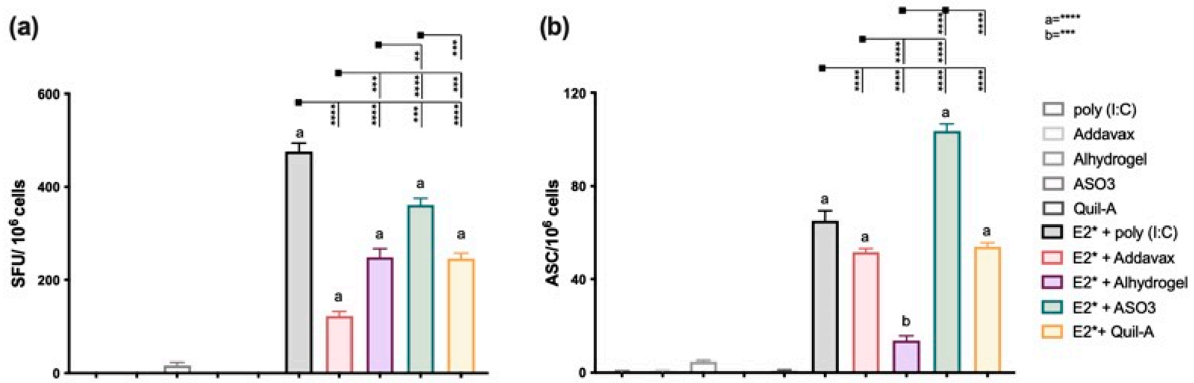

Figure S8. Immunization with E2* admixed with different adjuvants induces cellular immunity. C57BL/6 mice received 2 doses subcutaneously with 10µg of E2* protein in the presence of poly (I:C), Addavax, Alhydrogel, AS03 or QuilA. Fifteen days after boost, mice were euthanized and spleen and draining lymph nodes were removed. (**a**) Specific IFN-γ production was accessed by ELISpot against $E2_{231-250}$ peptide. SFU: spot forming units. (**b**) Draining lymph node cells were cultured with E2* for 18 hours to evaluate the number of ASC by ELISpot assay. a,b statistics of immunized groups compared to respective adjuvant control group. Statistical analysis was performed by One-way ANOVA followed by Tukey post-hoc test. Data represent mean ± SD. **p<0.01, ***p<0.001and ****p<0.0001.

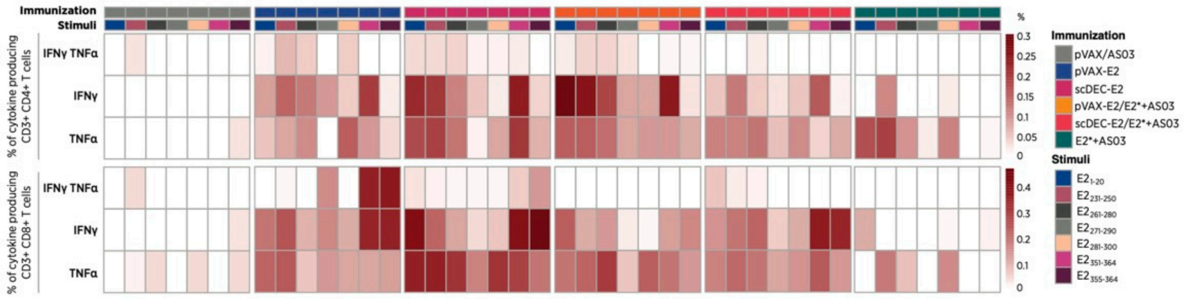

Figure S9. Immunization with vaccines containing E2CHIKV induces cytokine producing T cells. C57BL/6 mice were immunized as described in Figure 5. Fifteen days after boost, splenocytes were cultured for 12 hours in presence of specific E2-peptides. Cells were then stained with anti-CD3, -CD4, -CD8, -IFN-γ and -TNF-α and analyzed by flow cytometry. Heatmap showing the frequency of cytokine-producing T cells was calculated by subtracting the values from non-stimulated cells. Dark red represents higher production of the cytokines. Boolean combinations were created using the FlowJo platform. Data represent mean ± SD.

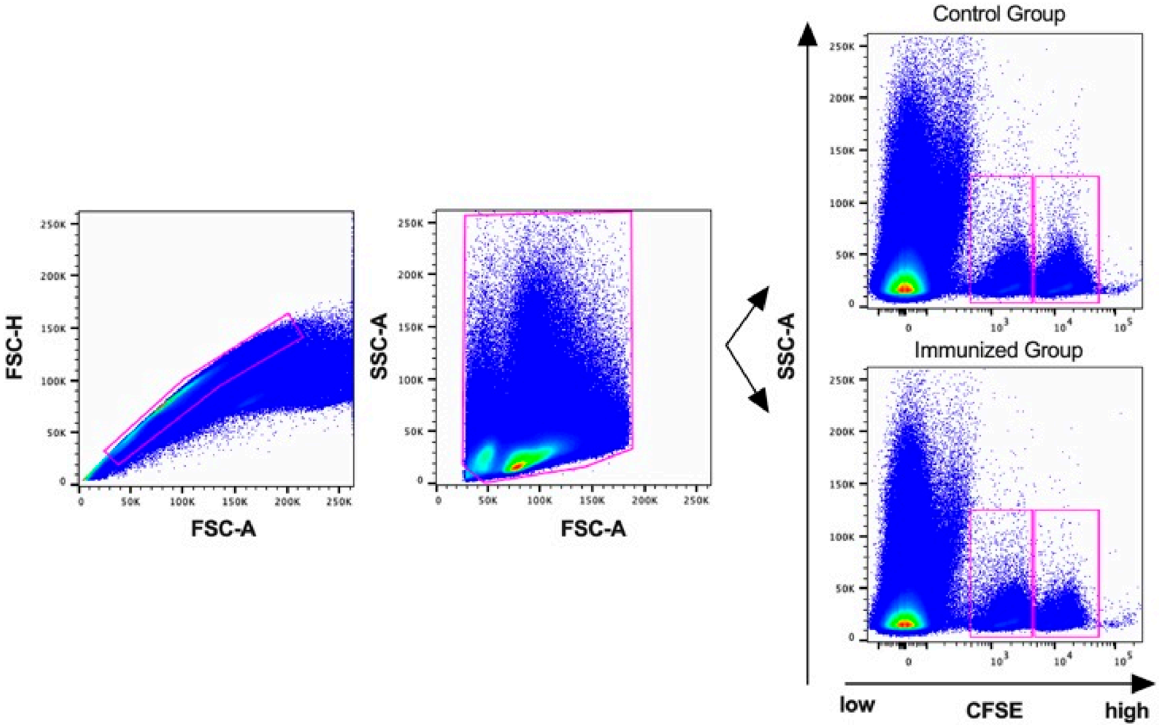

Figure S10. Flow cytometry gating strategy of *in vivo* cytotoxicity. C57BL/6 mice were immunized twice with 100 µg of the non-targeted pVAX-E2 DNA vaccine or a DC-targeted scDEC-E2 DNA vaccine followed by electroporation, or with 10 µg of E2* recombinant protein + AS03 subcutaneously. For heterologous DNAprime-protein boost, mice received one dose of DNA vaccine (pVAX-E2 or scDEC-E2) followed by one dose of recombinant E2* protein+ AS03. Control groups received the empty pVAX vector and poly AS03. Fifteen days after boost, *in vivo* $CD8^+$ T cell killing activity of target cells was evaluated after injection with syngeneic CFSE-labeled splenocytes pulsed with ($CFSE^{high}$) or without ($CFSE^{low}$) 1µg/mL of E2-specific peptide. One hundred thousand events were acquired inside the $CFSE^{low}$ gate.
